# Progress in Stability of Organic Solar Cells

**DOI:** 10.1002/advs.201903259

**Published:** 2020-04-22

**Authors:** Leiping Duan, Ashraf Uddin

**Affiliations:** ^1^ School of Photovoltaic and Renewable Energy Engineering University of New South Wales Sydney NSW 2052 Australia

**Keywords:** device engineering, International Summit on Organic Photovoltaic Stability guidelines, material design, organic solar cells, thin film photovoltaics

## Abstract

The organic solar cell (OSC) is a promising emerging low‐cost thin film photovoltaics technology. The power conversion efficiency (PCE) of OSCs has overpassed 16% for single junction and 17% for organic–organic tandem solar cells with the development of low bandgap organic materials synthesis and device processing technology. The main barrier of commercial use of OSCs is the poor stability of devices. Herein, the factors limiting the stability of OSCs are summarized. The limiting stability factors are oxygen, water, irradiation, heating, metastable morphology, diffusion of electrodes and buffer layers materials, and mechanical stress. The recent progress in strategies to increase the stability of OSCs is surveyed, such as material design, device engineering of active layers, employing inverted geometry, optimizing buffer layers, using stable electrodes and encapsulation materials. The International Summit on Organic Photovoltaic Stability guidelines are also discussed. The potential research strategies to achieve the required device stability and efficiency are highlighted, rendering possible pathways to facilitate the viable commercialization of OSCs.

## Graphical abstract

Herein, studies about the degradation mechanism and stability improving strategies of organic solar cells (OSCs) in the past few years are reviewed. The current inconsistency in the stability measurement and the importance of the International Summit on Organic Photovoltaic Stability standards are discussed, and outlooks and research directions in the stability of OSCs in the near future are proposed.

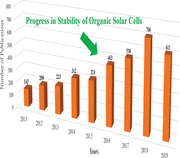

## 1 Introduction

As energy demands in the world keep increasing, developing renewable and sustainable energy has become a hot research topic in recent decades. Organic solar cells (OSCs) owning advantages of low cost, lightweight, flexible, semitransparent, and easy fabrication have emerged as a promising renewable energy technology.^[^ ^1^, ^2^, ^3^, ^4^, ^5^, ^6^ ^]^ In recent years, many studies focused on improving the power conversion efficiency (PCE) of OSCs by using strategies such as designing high‐performance donor and acceptor materials, employing ternary active layer, employing tandem device structure, optimizing carrier transport layers and optimizing the fabrication process.^[^ ^7^, ^8^, ^9^, ^10^, ^11^, ^12^, ^13^, ^14^, ^15^, ^16^, ^17^, ^18^, ^19^, ^20^ ^]^ As a result, the PCE of OSCs has boosted over 16% for single‐junction devices and over 17% for ternary and tandem devices.^[^ ^21^, ^22^, ^23^, ^24^, ^25^ ^]^

**Figure 1:**
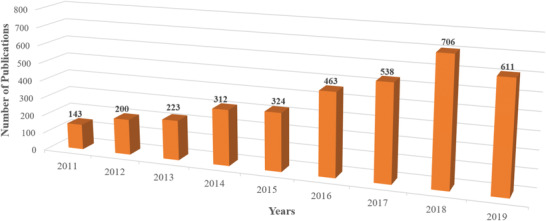
Publications trend in the stability of organic solar cells from 2011 to 2019. (Data obtained from Scopus.)

Nevertheless, as OSCs exhibited considerable potentials from the PCE perspective, the relatively low stability of the device is one of the main barriers for its future commercialization.^[^ ^26^ ^]^ OSCs suffer from degradations induced by several factors, and its performance can decrease rapidly in operation.^[^ ^27^ ^]^ Achieving long‐term stability in OSCs is still a significant challenge. Therefore, stability in OSCs has attracted much research attention in the past years. The number of publications about the stability of OSCs has kept increasing in recent years, as shown in **Figure** **1**. The degradation mechanism in OSCs has been widely investigated, and many novel strategies have been developed to enhance the stability of OSCs. To date, some studies have reported very stable OSCs. Brabec et al. proved a non‐fullerene acceptor based OSC with predicted lifetime approaching 10 years.^[^ ^28^ ^]^ More recently, Forrest et al. demonstrated an extremely stable class of thermally evaporated single‐junction OSCs with predicted outdoor lifetime up to 27 000 years.^[^ ^29^ ^]^ Thus, a Review on updated progress achieved in the stability of OSCs is needed for researchers to better understand and solve the stability bottleneck toward commercialization.

In this Review, we have focused on the limiting factors of stability studies of OSCs in the recent few years. First, we have summarized degradation and instability mechanisms in OSCs, such as inherent instability and burn‐in degradation. Second, we have discussed the current inconsistency in the stability measurement and emphasized the importance of International Summit on Organic Photovoltaic Stability (ISOS) standards. Third, we have discussed and summarized recently developed strategies to enhance the stability in OSCs. Finally, we have proposed outlooks and research directions in the stability of OSCs in the near future.

## 2 Mechanisms Behind Degradation/Instability

**Figure 2:**
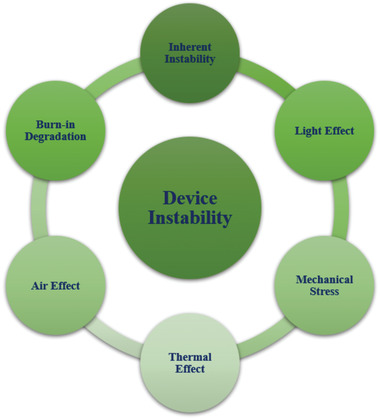
Schematic diagram of mechanisms behind the instability of OSCs. Each of these factors influence the degradation of OSCs.

Under the real operation condition, there are many factors related to the instability or degradation in OSCs. In this part, we have discussed those factors and the mechanism behind the instability from the perspectives of inherent stability, light instability under irradiation, thermal instability under heating, air instability under oxygen and water, burn‐in degradation, and mechanical instability, as shown in **Figure** **2**.

### 2.1 Inherent Instability

OSCs are highly sophisticated devices and show uncontrollable stability even without extrinsic degradation factors. There are two reasons for the inherent instability to occur: the morphology changes in the active layer and the diffusion of the carrier transport layer and electrode materials into the active layer.

**Figure 3:**
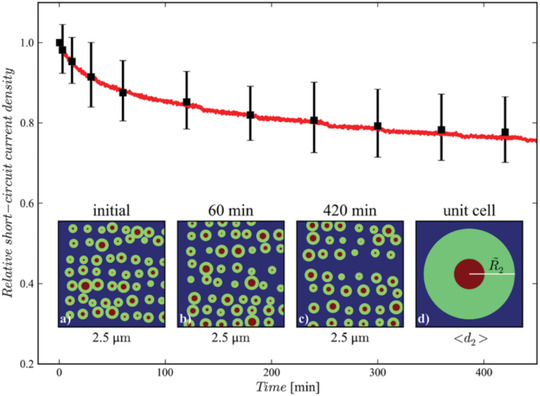
Simulated and measured short current density of OSCs with the donor and acceptor morphology evolution in the active layer from small domains to the larger one. Reproduced with permission.^[^ ^35^ ^]^ Copyright 2013, John Wiley and Sons.

The active layer of bulk heterojunction (BHJ) OSCs is the most critical component which possesses a nanoscale bicontinuous morphology with a mixed donor and acceptor phase.^[^ ^27^, ^30^, ^31^ ^]^ For obtaining the best performance, the donor and acceptor mixed morphology in the active layer requires an optimum condition to satisfy an efficient exciton dissociation and charge carriers transport process and limit the charge recombination process, simultaneously.^[^ ^32^ ^]^ However, the optimum morphology is usually not the thermodynamical steady case at equilibrium. As the optimum morphology formed, it will slowly evolve toward the equilibrium case and cause changes in the active layer.^[^ ^33^, ^34^ ^]^ Due to the high mobility of donor and acceptor materials and sometimes the structural incompatibilities between donor and acceptor, the phase separation of donor and acceptor is the result of the inherent morphology evolution in most of the cases.^[^ ^27^, ^35^, ^36^, ^37^ ^]^ Műller‐Buschbaum et al. proposed a morphology degradation model as for OSCs to describe the phase separation that the initially small domains in the active layer will evolve to the larger one, as shown in **Figure** **3**.^[^ ^35^ ^]^ Ma et al. analyzed the role of phase separation from the molecular packing and domain aggregation point of views and proved that the phase separation has profoundly adverse effects on the charge generation, transport, and extraction process in the active layer.^[^ ^38^ ^]^ They pointed out that the high stability of a blended system can result from its ideal miscibility that the initially optimized morphology is locally at the thermodynamic equilibrium.

Similar to donor and acceptor materials, the carriers transport layers and electrodes of OSCs also show mobility and result in an inherent instability. The use of widely applied electrodes, aluminium (Al) and indium tin oxide (ITO), can create a diffusion of Al and In atoms into the carriers transport layers and the active layer even without extrinsic factors.^[^ ^39^, ^40^, ^41^, ^42^, ^43^ ^]^ The use of widely applied carrier transport layer, poly(3,4‐ethylenedioxythiophene)‐poly(styrenesulfonate) (PEDOT:PSS), can also diffuse into the active layer.^[^ ^44^ ^]^ The diffusion of carriers’ transport layers and electrodes can change the energy levels of each layer and cause traps in the active layer, which accelerates the non‐radiative charge recombination.^[^ ^27^ ^]^

### 2.2 Light Effect

**Figure 4:**
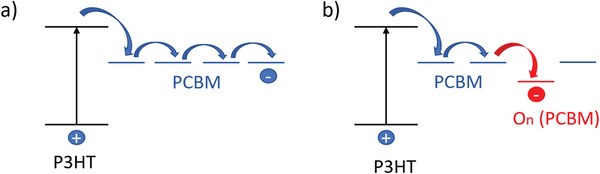
Demonstration of the energy transfer in a) fresh P3HT:PCBM film and b) how the deeper LUMO level of oxidized PCBM introduces trap states in photo‐degraded P3HT:PCBM film. Reproduced with permission.^[^ ^58^ ^]^ Copyright 2010, John Wiley and Sons.

Light‐induced degradation, also known as photo‐degradation, is one of the well‐known degradation mechanisms for OSCs. As OSCs are operating under illumination, the photo‐degradation issue is an inevitable barrier to fabricate real stable devices.^[^ ^45^ ^]^ The photochemical degradation and photophysical degradation in OSCs are the two main reasons for light instability.^[^ ^27^ ^]^ The photochemical degradation in OSCs is caused by the photo‐oxidation of the active layer and the carriers transport layer in most of the cases. In the active layer, both the donor and acceptor components can face a photo‐oxidation process under illumination, which changes the structures of donor and acceptor materials.^[^ ^46^ ^]^ The photo‐oxidation for polymer donors results from the generation of singlet oxygen and superoxide anions ($O_{2}^{-}$) under illumination.^[^ ^47^, ^48^ ^]^ Polymer donor poly [[4,8‐bis[(2‐ethylhexyl)oxy]benzo[1,2‐b:4,5‐b']dithiophene‐2,6‐diyl][3‐fluoro‐2‐[(2‐ethylhexyl)carbonyl]thieno[3,4‐b]thiophenediyl]] (PTB7) shows complete loss of fluorescence after being illuminated for 250 h.^[^ ^49^ ^]^ The photo‐oxidation for fullerene acceptors can cause the photopolymerization of fullerene molecules.^[^ ^50^ ^]^ The conventional fullerene acceptor bis(1‐[3‐(methoxycarbonyl)propyl]‐1‐phenyl)‐[6,6]C62 (PC_60_BM) can undergo a severe photodimerization after the exposure to sunlight.^[^ ^51^ ^]^ Kim et al. identified a three‐phase degradation process for non‐fullerene acceptors O‐IDTBR and O‐IDFBR which contains a first photoinduced conformational change, followed by photo‐oxidation and fragmentation and a complete chromophore bleaching at the final stage.^[^ ^52^ ^]^ The changed structures of donor and acceptors can cause the deterioration of its photovoltaic property and thus reduce the light absorption and charge generation in OSCs.^[^ ^46^, ^53^ ^]^ The changed structures can also tune the energy levels of donor and acceptors and change the energy level alignment, which causes the loss in performance.^[^ ^54^ ^]^ Moreover, the photo‐oxidation process can create sub‐bandgap state materials which induce traps and energetic disorder in the active layer to reduce the electron mobility and accelerate the non‐radiative recombination.^[^ ^55^, ^56^ ^]^ Wantz et al. demonstrated that the energy transfer from polymer to oxidized PCBMo*_x_* species could occur at the expense of the energy transfer to the pure PCBM.^[^ ^57^ ^]^ The oxidized PCBM species own a deeper lowest unoccupied molecular (LUMO) level than the pure PCBM which can act as electron traps in PCBM domains.^[^ ^58^ ^]^ **Figure** **4** shows the difference in the energy transfer between the fresh and photo‐degraded poly(3‐hexylthiophene‐2,5‐diyl) (P3HT):PCBM blend. It is worth to mention that the photo‐degradation of fullerene acceptors can also cause the formation of persistent free radicals, which can accumulate to decrease the device performance from another pathway.^[^ ^52^ ^]^ In addition to the photo‐oxidation of active layer, carrier transport layers also play a non‐negligible role in the photo‐degradation process. Carrier transport layers can take part in the photo‐oxidation process of the active layer in some cases. Ackerman et al. recently found that the widely used electron transport layer, zinc oxide (ZnO) nanoparticles, possesses the photocatalytic activity, which dramatically increases the photo‐oxidation rate of P3HT.^[^ ^59^ ^]^ The interface changes between the active layer and carrier transport layers are one of another cause contributes to the photo‐degradation. Kim et al. reported that the titanium dioxide nanoparticles bound with van der Waals interactive 3‐methyl‐2,4‐pentanedione (TNP‐Me) based electron transport layer can penetrate the photoactive layer after a light‐soaking process.^[^ ^60^ ^]^

Besides photo‐oxidation, other photochemical processes such as the photolysis of polymer donors and photo‐oligomerization of fullerene acceptors are also responsible for the photo‐degradation in OSCs.^[^ ^61^, ^62^ ^]^ Additionally, photophysical degradation is another pathway for photo‐degradation. Adachi et al. found that the photo‐degradation can induce charge accumulation in the devices and reported that accumulated charges are closely related to the decrease in device performance.^[^ ^63^, ^64^ ^]^

**Figure 5:**
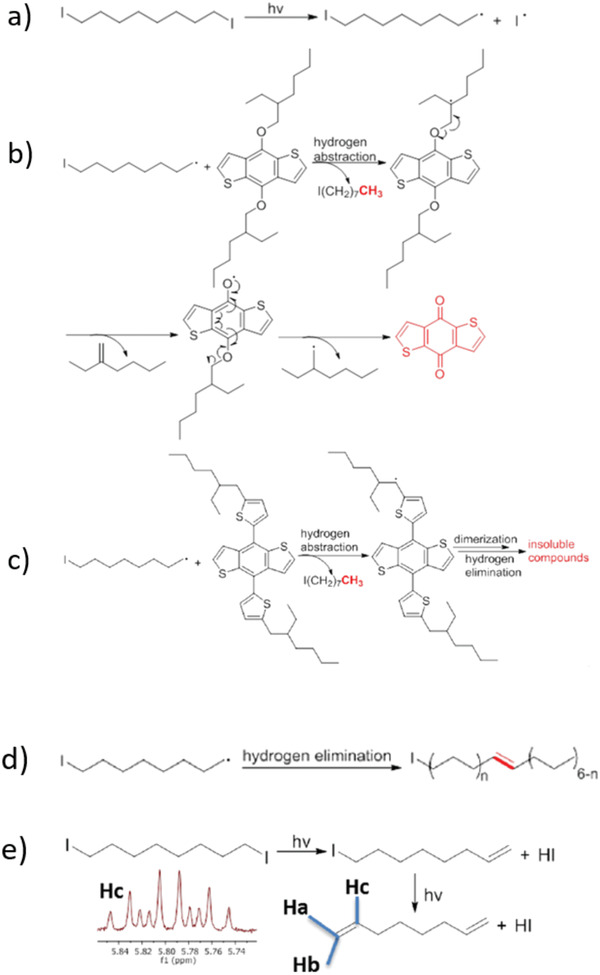
Possible degradation pathway after decomposition of DIO under UV light. A) Decomposition of DIO under UV light. DIO decomposition induced degradation pathways: b) hydrogen abstraction in E BDT monomer, c) ET BDT monomer. Hydrogen elimination d) in DIO with the formation of carbon‐centerd iodide radicals and e) in DIO with the formation of HI, an acid supported by the ^1^H NMR splitting pattern. Shown here, H_c_ is the vinylic proton at the 2 (or 7) position (H_c_) of the alkene radical present in solution. Reproduced with permission.^[^ ^73^ ^]^ Copyright 2019, Springer Nature.

Recently, many studies have focused on the photostability of OSCs based on the different light spectrum and ultraviolet (UV) light‐induced instability has attracted many research attentions.^[^ ^65^, ^66^, ^67^ ^]^ Photo‐degradation of OSCs was found more sensitive to the light at blue and UV wavelength range.^[^ ^65^, ^68^ ^]^ UV photons are the most energetic in the light spectrum. Nevertheless, they have less contribution to the energy conversion process in OSCs due to the absorption spectrum limitation of most of the organic materials.^[^ ^1^, ^69^ ^]^ The energetic UV photons were found to take the main contribution to the photo‐degradation in OSCs.^[^ ^70^, ^71^ ^]^ Someya et al. demonstrated that the photostability of OSCs was very different when tested with and without UV light.^[^ ^72^ ^]^ They found that the 2,5‐bis(3‐(2‐ethylhexyl‐5‐(trimethylstannyl)thiophen‐2‐yl)thiazolo[5,4‐d]thiazole‐2‐butyloctyl (PTzNTz‐BOBO):PC_71_BM based OSCs show a huge stability improvement when cutting the UV light in the illumination. On the other hand, UV light is harmful to the device stability since it can induce the decomposition of some light‐sensitive additives in the active layer.^[^ ^73^, ^74^ ^]^ Koster et al. recently reported that the widely used additives 1,8‐diiodooctane (DIO) could act as the photo‐acid under the UV light exposure.^[^ ^73^ ^]^ The UV irradiation can decompose the DIO residual in the active layer, which creates more electron traps to hinder the device performance. **Figure** **5** shows the possible degradation pathway in OSCs induced by the decomposition of DIO.

### 2.3 Thermal Effect

As solar cells are operating under continuous illumination, a relatively high working temperature with continuous heating is unavoidable. Unfortunately, many OSCs suffer from the thermal instability issue in the real world.^[^ ^75^, ^76^, ^77^, ^78^ ^]^ Since most materials applied in OSCs possess a much higher decomposition temperature than the operating temperature, the thermal instability of the device relate more to the physical degradation to a large extent.^[^ ^79^, ^80^, ^81^ ^]^

**Figure 6:**
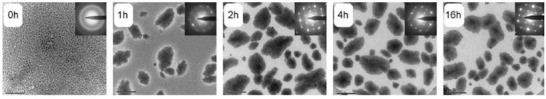
Thermal heating induced morphology changes in MDMO‐PPV:PCBM based films. Reproduced with permission.^[^ ^82^ ^]^ Copyright 2008, Elsevier.

In the active layer, the main reason for the thermal instability is that the morphology of the film is vulnerable and can change from its optimum condition under heating.^[^ ^35^, ^82^ ^]^ As mentioned, the optimum morphology in the active layer usually is not the thermodynamic equilibrium case that the morphology can slowly evolve to the equilibrium case without extrinsic factors. Heating which provides additional thermodynamic energy that can accelerate this morphology evolution even at a low temperature.^[^ ^34^, ^83^, ^84^ ^]^ Vanderzande et al. reported that the poly[2‐methoxy‐5‐(3′,7′‐dimethyloctyloxy)‐1,4‐phenylenevinylene] (MDMO‐PPV):PCBM based active layer shows severe morphology changes when continuously heating the film at 110 °C. The changes are positively related to the heating times, as shown in **Figure** **6**. On the other hand, polymer donors are proved to own enhanced mobility under a higher temperature than their glass transition temperature (*T*_g_).^[^ ^85^, ^86^ ^]^ Lidzey et al. found that the coarse phase separation can occur in the poly[*N*‐9'‐heptadecanyl‐2,7‐carbazole‐alt‐5,5‐(4ʹ,7ʹ‐di‐2‐thienyl‐2ʹ,1ʹ,3ʹ‐benzothiadiazole)] (PCDTBT):PC_71_BM based active layer when the applied temperature is higher than the glass transition temperature (*T*_g_) of the PCDTBT.^[^ ^87^ ^]^ For small molecular acceptors, the heating can create the aggregation and crystallization in the active layer.^[^ ^88^, ^89^, ^90^, ^91^ ^]^ Műller et al. demonstrated the nucleation and the crystallization of the PC_61_BM in the poly[2,3‐bis‐(3‐octyloxyphenyl)quinoxaline‐5,8‐diyl‐alt‐thiophene‐2,5‐diyl] (TQ1):PC_61_BM based active layer under the heating.^[^ ^91^ ^]^ The degree of the crystallization was found to be positively related to the applied temperature. When applied temperature is higher than the *T*_g_ of the blend film, the PC_61_BM shows distinct aggregation surrounded by a depletion zone.^[^ ^91^ ^]^ Those morphology changes can reduce the interface area between the donor and acceptor, which hinders the exciton separation and charge transport in the active layer.^[^ ^92^ ^]^

Besides the active layer, the heating can also cause degradation at the contact interface and in carrier transport layers. The morphology changes in the active layer can change the adhesion between the active layer and carrier transport layers and create the barrier for electron extraction.^[^ ^93^ ^]^ Uddin et al. recently revealed that the thermal degradation in both PTB7‐Th:PC_71_BM based fullerene and PTB7‐Th:3,9‐bis(2‐methylene‐(3‐(1,1‐dicyanomethylene)‐indanone))‐5,5,11,11‐tetrakis(4‐hexylphenyl)‐dithieno[2,3‐d:2′,3′‐d′]‐s‐indaceno[1,2‐b:5,6‐b′]dithiophene based non‐fullerene based devices can cause severe deterioration in charge extraction process and result in high leakage current.^[^ ^94^ ^]^ Carrier transport layers such as PEDOT:PSS can show decreased electrical conductivity under heating.^[^ ^95^ ^]^ On the other hand, the heating can also accelerate the metal atom diffusion process caused by metal electrodes to decrease the device performance as most diffusion processes are temperature dependent.^[^ ^96^, ^97^, ^98^ ^]^

### 2.4 Air Effect

**Figure 7:**
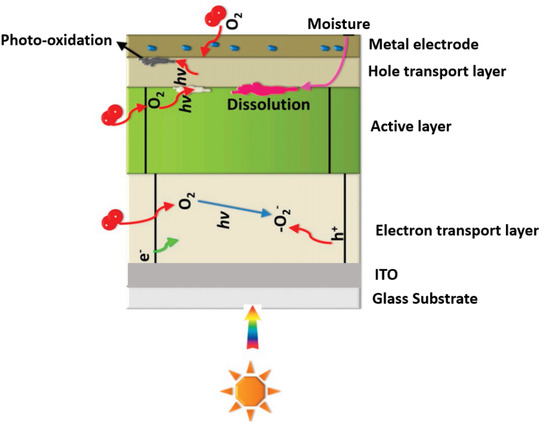
Schematic diagram of the air effect, including oxygen and moisture effect on OSCs. Adapted with permission.^[^ ^280^ ^]^ Copyright 2016, The Royal Society of Chemistry.

Air instability is another issue for most of OSCs as the ingress of ambient oxygen and water can be detrimental to the device performance. Ambient oxygen and water can diffuse through the whole device and cause severe physical and chemical degradation in electrodes, carrier transport layers, and active layers of OSCs. Oxygen and water permeation can cause oxidation to occur in metal electrodes such as Al and calcium (Ca) with low function, which creates an electrically insulating metal oxide layer to obstacle the charge transport and extraction.^[^ ^99^ ^]^ Moreover, defects can form at the interface between the electrodes and carrier transport layers after the oxidation, which acts as pinholes to reduce the device performance.^[^ ^39^, ^100^ ^]^ Some carrier transport layers are sensitive to ambient oxygen and water. The widely used hole transport layer PEDOT:PSS can show acidity and hygroscopicity in air.^[^ ^101^, ^102^ ^]^ A schematic diagram of the air effect on OSCs has been shown in **Figure** **7**. As shown in Figure 7, the diffusion of oxygen into the device can result in photo‐oxidation of the active layer materials with the combination of light irradiance. The diffusion of oxygen into the carrier transport layer such as the TiO_2_ based electron transport layer can cause oxygen defects which can trap the electron and hinder the current extraction. On the other hand, the diffusion of moisture into the device can cause the dissolution of the device materials to hinder the device performance.

Friedel et al. recently proved that carrier transport layers PEDOT:PSS and molybdenum trioxide (MoO_3_) could absorb water in ambient and cause a loss in the interfacial contact area. Both of these two types of layer showed a swelling/shrinking reaction in the air, which induced a micro‐delamination and severely decreased the photocurrent in the device.^[^ ^103^ ^]^ Zhu et al. found that the PEDOT:PSS can react with Poly[(2,6‐(4,8‐bis(5‐(2‐ethylhexyl)thiophen‐2‐yl)‐benzo[1,2‐b:4,5‐b′]dithiophene))‐alt‐(5,5‐(1′,3′‐di‐2‐thienyl‐5′,7′‐bis(2‐ethylhexyl)benzo[1′,2′‐c:4′,5′‐c′]dithiophene‐4,8‐dione)]:ITIC based active layer and decrease the built‐in potential in the device.^[^ ^104^ ^]^ For active layers, as mentioned, the oxygen and water can take part in the photo‐oxidation process of the donor and acceptor materials which changed their chemical structures and tuned their energy levels, absorption spectrums, and electron mobilities.^[^ ^58^ ^]^ Liu et al. found that exposure to oxygen gas can change the electronic structure of non‐fullerene acceptors ITIC and o‐IDTBR. They also showed that water vapor exposure could slightly change the highest occupied molecular orbital (HOMO) level of these two acceptors.^[^ ^105^ ^]^ On the other hand, the permeation of oxygen and water can cause morphology changes in the active layer. Cooperating with water, the fullerene acceptor PC_61_BM can move and show excessive aggregation behavior.^[^ ^106^ ^]^ PBDB‐T:ITIC based active layer can show a continuous vertical phase separation process when aging in the air.^[^ ^104^ ^]^ Those morphology changes can cause the reduction of interface area between the donor and acceptor and induce a poor exciton separation and charge transport process.

The oxygen and water can also cause oxygen doping in the device. The oxidation of the active layer and the permeation of oxygen atoms can increase the hole concentration in the device which reduces the density of deeper traps for electrons and results in the reduction of the fill factor (FF) and open‐circuit voltage (*V*_oc_) of OSCs.^[^ ^107^, ^108^ ^]^ Vaynzod et al. recently proved that the oxygen‐induced p‐type doping occurs even without the chemical oxidation of materials which is the leading cause for the performance degradation in poly[(5,6‐difluoro‐2,1,3‐benzothiadiazol‐4,7‐diyl)‐alt‐(3,3ʹʹʹ‐di(2‐octyldodecyl)‐2,2ʹ,5ʹ,2ʹʹ,5ʹʹ,2ʹʹʹ‐quaterthiophen‐5,5ʹʹʹ‐diyl)] (PffBT4T‐2OD):PC_71_BM based OSCs.^[^ ^109^, ^110^ ^]^ It is worth to mention that oxygen and water always simultaneously degrade the device in reality.^[^ ^111^ ^]^ OSCs degraded in the air with different humidities can show an apparent difference.^[^ ^112^, ^113^ ^]^

### 2.5 Burn‐In Degradation

**Figure 8:**
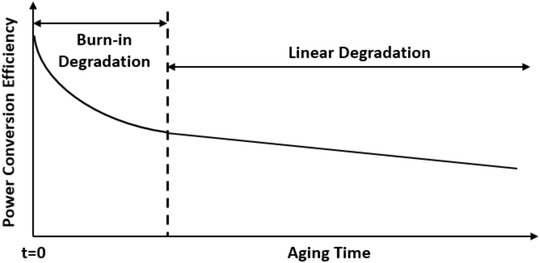
Typical lifetime curve for OSCs with two degradation regimes, including burn‐in degradation and linear degradation. Reproduced with permission.^[^ ^26^ ^]^ Copyright 2016, John Wiley and Sons.

In the real world, all of those factors for instability simultaneously exist in OSCs. From the lifetime perspective, most OSCs can show two degradation regimes, as shown in **Figure** **8**, which includes an initial sharp performance degradation followed by slow linear performance degradation.^[^ ^114^, ^115^, ^116^ ^]^ The initial sharp performance degradation also called as “burn‐in” degradation, can contribute to the significant part of the performance loss. The state‐of‐the‐art PBDB‐T:ITIC based non‐fullerene and PTB7‐Th:PC_71_BM based fullerene devices can both lose over 30% of their initial PCE in few hours under the real operation condition.^[^ ^117^, ^118^ ^]^ The origin of burn‐in degradation is used to be a controversial research topic. Researchers listed many possible reasons such as the fullerene dimerization, the increased trap mediated charge recombination, the broad polydispersity of polymer donors, and organic or inorganic impurities in the film.^[^ ^115^, ^117^, ^119^, ^120^, ^121^, ^122^ ^]^ However, many recent studies revealed that the morphology changes in the active layer are the real root reason for the burn‐in loss.^[^ ^37^, ^123^, ^124^, ^125^ ^]^ Brabec et al. found that the spinodal demixing of the donor and acceptor is the leading cause for the burn‐in degradation in PTB7‐Th:PCBM based OSCs.^[^ ^37^ ^]^ They also observed the same phenomenon in PffBT4T‐2OD:PC_71_BM based OSCs.^[^ ^124^ ^]^ For OSCs, both the exterior factors of light and heat can be the reason to cause the morphology change in burn‐in degradation.^[^ ^94^ ^]^ However, many recent studies proved that heat can be actually the dominant reason. For example, Chen et al. recently implied that the heat‐induced aggregation in the active layer is the leading cause for the dramatic burn‐in degradation in the P3HT:PC_61_BM system.^[^ ^126^ ^]^ Herein, we have agreed a possible explanation for the burn‐in behavior in OSCs based on the morphology change. As mentioned, the as‐cast optimum morphology in the active layer is usually not at the thermodynamic equilibrium case and can slowly evolve to the equilibrium case. Extrinsic degradation factors in the real world can cause morphology changes and accelerate this morphology evolution which presents as a fast degradation at the initial stage. When the morphology evolved close to the equilibrium, the rate of evolution can slow down and present as the slow linear degradation.

### 2.6 Mechanical Stress Effect

**Figure 9:**
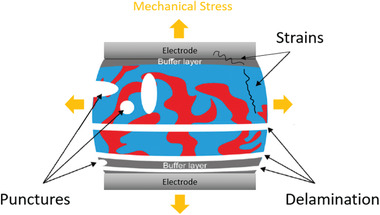
Types of mechanical degradation induced by mechanical stress, including the stress caused strains, punctures, and delamination. Adapted with permission.^[^ ^27^ ^]^ Copyright 2016, The Royal Society of Chemistry.

Mechanical instability which is induced by mechanical stress is unavoidable in OSCs since that mechanical stress can occur in the roll‐to‐roll fabrication, installation, and transportation of the devices.^[^ ^127^ ^]^ Factors in real weather condition such as rain, wind, and hail can also cause mechanical stress to devices.^[^ ^128^ ^]^Moreover, OSCs applications like flexible solar cells and wearable solar cells can even face more mechanical stress.^[^ ^129^, ^130^, ^131^, ^132^, ^133^ ^]^ It is worth to mention that tensile mechanical pressure is a more dominant cause in those stress‐induced degradation mechanisms than the compression vertical pressure.^[^ ^134^ ^]^ There are several types of degradation that can occur in OSCs under mechanical stress, as shown in **Figure** **9**. First, the linear mechanical tension can create strains of organic materials in the active layer which changed the morphology and decreased the device performance.^[^ ^135^ ^]^ Second, mechanical stress can cause delamination of the active layer, carrier transport layers, and electrodes. The delamination occurred depending on the adhesion energy of each layer that can reduce the area of the contact interface and hinder the charge transport and extraction.^[^ ^136^ ^]^ Third, mechanical stress may cause punctures and cracks in every layer of the devices, which facilitates the ingress of oxygen and water in the devices and thus accelerates the degradation.^[^ ^137^, ^138^ ^]^ Fourth, the lateral strains can be another degradation reason. As the critical strain value of materials in each layer is different, lateral strain can first cause degradation in the relative brittle layer such as ITO electrode layer under the same strain value to degrade the device performance.^[^ ^139^, ^140^ ^]^ Using the material with high strain value to replace the brittle material can be an effective way to prevent the lateral strains.

## 3 Stability Measurements

Proper testing and correct rating the stability is an indispensable element for understanding, comparing, and improving the stability of OSCs. In this part, we have discussed the miscellaneous stability measurements and emphasized the ISOS test guidelines.

### 3.1 Miscellaneous Stability Measurements

In the past few years, there are many studies that focused on the measurement and improvement of the stability in OSCs. However, their stability test conditions, test duration, and lifetime baseline are miscellaneous. It is hard to enable comparison of works between different laboratories, neither to confirm the effectiveness of their results.

According to stability studies over the past few years, we have summarized various stability test conditions based on each type of stability measurement. In the light stability measurement, there are three typical test conditions usually used: 1) under simulated one‐sun illumination in the air;^[^ ^141^ ^]^ 2) under simulated one‐sun illumination in the inert atmosphere;^[^ ^142^ ^]^ and 3) under simulated one‐sun illumination without or with weak UV light component.^[^ ^143^, ^144^ ^]^ The test with and without air and UV light can cause results to show a significant difference. The dominant degradation mechanism under those three test conditions may differ. Moreover, the temperature control during the light stability test from different works is also varied. Without temperature control, controlled at room temperature, and controlled at predicted working temperature (usually 80 °C) can also cause the results to vary. In the thermal stability measurement, there are two categories of test conditions: 1) under high‐temperature condition above 100 °C^[^ ^145^ ^]^ and 2) under low‐temperature condition below 100 °C.^[^ ^83^ ^]^ As mentioned, the application of high temperature can cause nucleation and crystallization of organic materials, while the application of low temperature cannot. In the storage stability test, there are three widely used conditions: 1) stored in the air without encapsulation;^[^ ^146^ ^]^ 2) stored in the air with encapsulation and;^[^ ^147^ ^]^ 3) stored in an inert atmosphere.^[^ ^148^ ^]^ Moreover, humidity control for different storage stability tests also varies. As mentioned, the degradation of OSCs in the air with different humidity can differ a lot.^[^ ^112^, ^113^ ^]^ In the mechanical stability measurement, the bending test is the commonly used method to do the stability measurement.^150^ However, there is still no consistent standard for the bending test to evaluate OSCs. Different works tested their devices with different bending radius and cycles.^[^ ^149^, ^150^ ^]^ Some studies also implied that the applied strain values could be more meaningful than just the bending radius since different film thickness and Young's modulus can result in different applied strain values to devices even though the same bending radius.^[^ ^151^, ^152^, ^153^ ^]^ Moreover, for the flexible electronic device, it was found that locating devices at neutral strain position can also reduce the strain value.^[^ ^154^, ^155^, ^156^, ^157^ ^]^

On the other hand, the duration of the stability test and the lifetime baseline from different works are also inconsistent. The test duration can vary from a few hours to a few months.^[^ ^158^, ^159^ ^]^ The way to report the lifetime of the devices are miscellaneous. Most of the works only reported the performance of the device at the end of the degradation test.^[^ ^73^, ^160^, ^161^ ^]^ Only a few works used the lifetime baselines such as T_80_ and T_50,_ which represent the time when devices degraded by 20% and 50% of the initial performance.^[^ ^28^, ^162^, ^163^ ^]^

### 3.2 ISOS Test Guidelines

To reach the consistency in the stability test of OSCs, ISOS guidelines were established in 2011.^[^ ^164^ ^]^ ISOS refers to the ISOS which is considered as a milestone in the research field of OSCs. ISOS guidelines set instructions for the stability test of OSCs under different conditions such as dark, indoor weathering, outdoor, and thermal cycling. **Table** **1** listed a summary for ISOS test guidelines. As shown in Table 1, ISOS guidelines set three levels of the testing, which vary in complexity and equipment requirements to meet the demand from different research laboratories. The higher‐level selected results in the higher accuracy of the test.

**Table 1:** Summary of ISOS test guidelines for different test conditions. Reproduced with permission.^[^ ^164^ ^]^ Copyright 2011 Elsevier

Three levels						
Basic (level 1): “Handheld” measurements using the simplest equipment and few conditions.						
Intermediate (level 2): Fixed conditions and protocols suited for most labs.						
Advanced (level 3): Standardized tests applied in certified labs. Extended range of parameters to monitor etc.						
Test type	Dark			Outdoor		
Test ID	ISOS‐D‐1 shelf	ISOS‐D‐2 high temp. storage	ISOS‐D‐3 damp heat	ISOS‐O‐1 outdoor	ISOS‐O‐2 outdoor	ISOS‐O‐3 outdoor
Light source	None	None	None	Sunlight	Sunlight	Sunlight
Temp.^a)^	Ambient	65/85 °C	65/85 °C	Ambient	Ambient	Ambient
Relative humidity (RH)^a)^	Ambient	Ambient (low)	85%	Ambient	Ambient	Ambient
Environment^a)^	Ambient	Oven	Env. chamber	Outdoor	Outdoor	Outdoor
Characterization light source	Solar simulator or sunlight	Solar simulator	Solar simulator	Solar simulator	Sunlight	Sunlight and solar simulator
Load^b)^	Open circuit	Open circuit	Open circuit	MPP or open circuit	MPP or open circuit	MPP

Test type	Laboratory weathering testing			Thermal cycling		
Test ID	ISOS‐L‐1 laboratory weathering	ISOS‐L‐2 laboratory weathering	ISOS‐L‐3 laboratory weathering	ISOS‐T‐1 thermal cycling	ISOS‐T‐2 thermal cycling	ISOS‐T‐3 thermal cycling
Light source	Simulator	Simulator	Simulator	None	None	None
Temp.^a)^	Ambient	65/85 °C	65/85 °C	Between room temp. and 65/85 °C	Between room temp. and 65/85 °C	−40 to +85 °C
Relative humidity (RH)^a)^	Ambient	Ambient	Near 50%	Ambient	Ambient	Near 55%
Environment/setup	Light only	Light and temp.	Light, temp., RH	Hot plate/oven	Oven/env. chamb.	Env. chamb.
Characterization light source	Solar simulator	Solar simulator	Solar simulator	Solar simulator or sunlight	Solar simulator	Solar simulator
Load^b)^	MPP or open circuit	MPP or open circuit	MPP	Open circuit	Open circuit	Open circuit

a) The ambient conditions are defined as 23 °C/50% RH in general, and 27 °C/65% RH accepted in tropical countries according to ISO291 (2008): Plastics—Standard atmospheres for conditioning and testing

b) Open circuit refers to a simply disconnected device or device connected to a source meter set to 0 current.

Unfortunately, during the past few years, only a limited number of stability studies used and specified the ISOS guidelines in their works.^[^ ^160^, ^165^, ^166^, ^167^ ^]^ Stability tests in many works which were conducted in an inert atmosphere or with a light source with poor UV performance are incompatible with the guidelines.^[^ ^144^, ^162^, ^168^ ^]^ Recently, Krebs et al. revealed the limitations of conducting stability test in an inert atmosphere and without UV light.^[^ ^169^ ^]^ They pointed out that the test in an inert atmosphere cannot provide a real insight into the stability potential under real operating condition, and it is not suitable for comparative stability studies. For the stability test without UV light, they stated that such a test might be useful for imitating the indoor application. As UV light is a critical component of real sunlight that can cause primary degradation, they suggested researchers follow the ISOS guidelines, if they need to investigate the stability of their devices in the real environment. It is interesting to find that some studies have claimed that their devices could get rid of the light‐induced burn‐in degradation based on the light stability test without the UV light.^[^ ^143^, ^144^ ^]^ As mentioned, the UV light can contribute to the primary degradation mechanism behind the light effect, we do not recommend the stability test that rationally ignores the UV light. To date, although there are many newly reported stability studies and many newly developed stabilities improving strategies, it is difficult to make a census discussion over those works. Researchers tend to bias select the stability test procedure to make their results convincible. For instances, the high‐temperature aging test up to 150 °C is unnecessary since the real operating temperature of solar cells can barely exceed 85 °C.^[^ ^170^ ^]^ Thus, we propose that the stability test with a high temperature over 100 °C can be useful for organic materials, while it is unnecessary for the lifetime test of the OSC. The degradation induced by the high‐temperature condition over 100 °C may not occur in OSCs during their operation in the real case. Although ISOS test guidelines also have some limitations such as that it uncovers the mechanical stability and its outdoor test may be affected by the geographic and climatic conditions.^[^ ^171^ ^]^ It is currently the most consensus and comprehensive protocol for the stability test. Herein, we highly recommend ISOS test guidelines for all researchers to use in future.

## 4 Progress in Enhancing Stability

In this part, we have focused on the progress in the enhancement of the stability of OSCs. We have summarized and discussed the stability‐enhancing strategies developed in the past few years. Those strategies are divided into four main groups: the material design and selection, device engineering, processing techniques, and encapsulation. All strategies are listed in **Table** 2, 3, 4, 5, 6, 7, 8, 9, 10 based on a different part. In these tables, test conditions are specified based on exterior factors, including light, heat, and air with humidity. Tests referred to ISOS guidelines are directly specified by the ISOS code. The “w” represents “with,” “w/o” represents “without,” and “na” represents “ not available” in the table. Test duration and photovoltaic parameters of the device are also given in the table. The photovoltaic parameters after the test duration if available are shown in the bracket and maintained PCE ratio after the test represented as PCE/PCE_0_ is shown as a separate column.

**Table 2:** The photovoltaic parameters of devices in stability enhancement strategies based on donor materials design and selection

	Test conditions										
Active layer	Light	Heat	Air (relative humidity)	Duration	**Strategy**	*V*_oc_ [V]	*J*_sc_ [mA cm^−2^]	FF	PCE [%]	PCE/PCE_0_ [%]	Ref.
BDTT‐TR:PC_70_BM	w/o	120 ^°^C	na	80 h	**w/o**	0.93	11.8	0.68	7.44	20	^[^ ^177^ ^]^
					**Change donor to BDTT‐O‐TR**	0.90	11.0	0.66	6.50	20	
					**Change donor to BDTT‐S‐TR**	0.97	13.5	0.71	9.20	35	
F8T2Ox1:PC_61_BM	na	na	w (na)	24 days	**w/o**	0.87	3.7	0.39	1.27	45	^[^ ^178^ ^]^
					**Change donor to crosslk‐F8T2Ox1**	0.94	3.0	0.37	1.06	86	
PPDT2FBT:PC_71_BM	na	120 ^°^C	w (na)	40 h	**w/o**	0.82	12.2	0.70	6.95	58	^[^ ^179^ ^]^
					**Change donor to PPDT2FBT‐V_10_**	0.80	13.7	0.70	7.63	100	
PTB7‐Th:PC_71_BM	w	Room temp.	w/o	2 h	**w/o**	0.80	15.1	0.69	8.62	65	^[^ ^158^ ^]^
					**Change donor to PTB7**	0.80	10.1	0.56	4.54	90	
PBDTTT‐C‐T:PC_71_BM					**w/o**	0.84	11.3	0.55	5.21	48	
					**Change donor to PBDTTT‐C**	0.74	12.3	0.46	4.17	84	
PBDTTT‐ET:PC_70_BM	w	na	na	2 h	**w/o**	0.70	13.4	0.62	5.80	60	^[^ ^73^ ^]^
					**Change donor to PBDTTT‐E**	0.65	13.1	0.68	5.00	80	
COOP‐1HT‐BDT:PC_71_BM	w	na	w/o	320 h	**w/o**	0.94	1.5	0.36	0.52	52	^[^ ^180^ ^]^
					**Change donor to COOP‐2HT‐BDT**	1.00	5.8	0.54	3.13	73	
					**Change donor to COOP‐3HT‐BDT**	0.91	7.0	0.57	3.64	81	
					**Change donor to COOP‐4HT‐BDT**	0.90	8.3	0.69	5.14	85	
PBDTTT‐C:PC_71_BM	w	Room temp.	w (50–60%)	1 h	**w/o**	0.71 (0.69)	14.6 (13.5)	0.56 (0.43)	5.80 (4.00)	69	^[^ ^141^ ^]^
					**Change donor to PTB7**	0.74 (0.74)	14.7 (13.8)	0.69 (0.64)	7.50 (6.50)	87	
PBDTTT‐C‐T:PC_71_BM					**w/o**	0.74 (0.75)	16.8 (11.6)	0.55 (0.38)	6.90 (3.30)	48	
					**Change donor to PTB7‐Th**	0.77 (0.77)	17.6 (17.4)	0.65 (0.58)	8.80 (7.90)	90	
P3HT:PCBM	w	na	w (na)	150 h	**w/o**	na	na	na	na	22	^[^ ^181^ ^]^
PTB7‐Th:PCBM					**w/o**	na	na	na	na	0	
					**Change donor to P3HT‐b‐PTB7‐Th**	0.58	9.8	0.62	3.5	90	
PTzBI‐Si_L_:N2200	w/o	w/o	w/o	1000 h	**w/o**	0.85	13.7	0.72	8.4	85	^[^ ^182^ ^]^
					**Change donor to PTzBI‐Si_H_**	0.85	17.2	0.78	11.2	98	
	w/o	80 ^°^C	w/o	1000 h	**w/o**	0.85	13.7	0.72	8.4	81	
					**Change donor to PTzBI‐Si_H_**	0.85	17.2	0.78	11.2	92	

**Table 3:** The photovoltaic parameters of devices in stability enhancement strategies based on acceptor materials design and selection

	Test conditions										
Active layer	Light	Heat	Air (relative humidity)	Duration	**Strategy**	*V*_oc_ [V]	*J*_sc_ [mA cm^−2^]	FF	PCE [%]	PCE/PCE_0_ [%]	Ref.
PBDTTS‐TTffBT:PC_71_BM	w/o	100 ^°^C	na	200 min	**w/o**	0.85	14.8	0.71	8.9	0	^[^ ^190^ ^]^
					**Change acceptor to di‐PBI**	0.92 (0.90)	10.9 (9.2)	0.65 (0.55)	6.5 (4.6)	70	
P3HT:PCBM	na	120 ^°^C	na	100 h	**w/o**	0.58	10.2	0.56	3.4	34	^[^ ^191^ ^]^
					**Change acceptor to SF‐HR**	1.00	8.2	0.49	4.0	83	
P3HT:PCBM	w	na	w (na)	100 h	**w/o**	0.58	10.2	0.56	3.4	28	
					**Change acceptor to SF‐HR**	1.00	8.2	0.49	4.0	72	
W‐CN:PC_70_BM	n/o	100 ^°^C	w (na)	24 h	**w/o**	0.99	10.1	0.59	5.9 (0.4)	6	^[^ ^192^ ^]^
					**Change acceptor to ITIC**	0.95	9.1	0.45	3.9 (2.9)	74	
					**Change acceptor to IDIC**	0.90	11.6	0.50	5.3 (5.0)	95	
PffBT4T‐2OD:PC_71_BM	w	50 ^°^C	w (na)	60 h	**w/o**	0.76	20.5	0.01	10.9	80	^[^ ^144^ ^]^
					**Change acceptor to IDTBR**	1.08	14.7	0.60	9.5	100	
P3HT:PCBM	w	na	w/o	2000 h	**w/o**	0.54 (0.51)	9.3 (6.8)	0.60 (0.58)	3.0 (2.0)	67	^[^ ^143^ ^]^
					**Change acceptor to IDTBR**	0.72 (0.72)	12.6 (13.1)	0.67 (0.62)	6.1 (5.9)	97	
PTB7‐Th:PC_71_BM	w	na	w/o	100 h	**w/o**	0.78	15.9	0.66	8.5	20	^[^ ^142^ ^]^
					**Change acceptor to EHIDTBR**	1.01	18.2	0.56	10.5	70	
PBDB‐T:PCBM	w/o	w/o	w/o	300 h	**w/o**	0.87	10.3	0.65	5.8	90	^[^ ^193^ ^]^
					**Change acceptor to N2200**	0.86	11.1	0.64	6.1	95	
PBDB‐T:PCBM	w/o	80 °C	w/o	300 h	**w/o**	0.87	10.3	0.65	5.8	52	
					**Change acceptor to N2200**	0.86	11.1	0.64	6.1	98	
PBDB‐T:PCBM	w/o	Room temp.	w (20–30%)	300 h	**w/o**	0.87	10.3	0.65	5.8	60	
					**Change acceptor to N2200**	0.86	11.1	0.64	6.1	90	
PTB7‐Th:PC_71_BM	w/o	na	w (na)	1000 h	**w/o**	0.82 (0.80)	13.4 (12.7)	0.51 (0.46)	5.6 (4.7)	61	^[^ ^194^ ^]^
					**Change acceptor to IDT(TCV)2**	0.47 (0.48)	10.0 (10.0)	0.58 (0.61)	2.7 (2.9)	110	
					**Change acceptor to IDTT(TCV)2**	0.60 (0.61)	11.3 (10.9)	0.52 (0.54)	3.5 (3.6)	103	
PBDB‐T:ITIC	na	150 ^°^C	na	12 h	**w/o**	na	na	na	na	65	^[^ ^195^ ^]^
					**Change acceptor to DF‐PCIC**	0.91	15.7	0.72	10.1	88	
P3HT:PCBM	w/o	130 ^°^C	na	300 min	**w/o**	0.59	8.2	0.68	3.3	10	^[^ ^196^ ^]^
					**Change acceptor to TQT‐C60**	0.61	6.2	0.44	1.7	95	
					**Change acceptor to TBTT‐C60**	0.61	6.2	0.58	2.2	95	
					**Change acceptor to TBST‐C60**	0.60	7.1	0.58	2.5	95	
PBDB‐T:ITIC	w	Room temp.	w (na)	120 min	**w/o**	0.88	13.4	0.64	7.5	85	^[^ ^197^ ^]^
					**Change acceptor to IT‐M**	0.92	12.9	0.60	7.8	88	
					**Change acceptor to IT‐F**	0.72	14.3	0.60	6.2	78	
PBDB‐T‐2F:ITIC‐Cl‐m	w	Room temp.	w/o	200 h	**w/o**	0.90	18.0	0.67	10.9	75	^[^ ^198^ ^]^
					**Change acceptor to ITIC‐Cl‐*δ***	0.90	18.3	0.70	11.5	80	
					**Change acceptor to ITIC‐Cl‐r**	0.93	18.9	0.74	13.0	86	
	w/o	80 ^°^C	w/o	200 h	**w/o**	0.90	18.0	0.67	10.9	78	
					**Change acceptor to ITIC‐Cl‐*δ***	0.90	18.3	0.70	11.5	82	
					**Change acceptor to ITIC‐Cl‐r**	0.93	18.9	0.74	13.0	90	

**Table 4:** The photovoltaic parameters of devices in stability enhancement strategies based on electron transport materials design and selection

	Test conditions										
Active layer	Light	Heat	Air (relative humidity)	Duration	**Strategy**	*V*_oc_ [V]	*J*_sc_ [mA cm^−2^]	FF	PCE [%]	PCE/PCE_0_ [%]	Ref.
P3HT:PC_61_BM	w/o	80 °C	w/o	160 h	**w/o (LiF as ETL)**	0.63	9.2	0.50	2.9	65	^[^ ^199^ ^]^
					**Change ETL to C‐CQDs**	0.63	9.4	0.50	3.0	82	
PTB7‐Th:PC_71_BM	w/o	na	w (na)	7 days	**w/o (ZnO as ETL)**	0.80 (0.59)	16.1 (7.8)	0.68 (0.40)	8.7 (1.8)	21	^[^ ^200^ ^]^
					**Change ETL to H1**	0.79 (0.73)	15.8 (14.3)	0.63 (0.52)	7.8 (5.4)	69	
					**Change ETL to H2**	0.77 (0.69)	15.6 (13.8)	0.54 (0.51)	6.5 (3.9)	60	
PTB7‐Th:PC_71_BM	na	na	w (na)	30 days	**w/o (Ca as ETL)**	0.80	14.6	0.69	8.3	36	^[^ ^166^ ^]^
					**Change ETL to PAMPS‐Na**	0.80	15.8	0.70	9.2	77	
PTB7‐Th:PC_71_BM	w	Room temp.	na	1000 h	**w/o (TNP as ETL)**	0.71(0.55)	16.4 (4.3)	0.57 (0.39)	6.6 (0.9)	14	^[^ ^60^ ^]^
					**Change ETL to TNP‐Ph**	0.82 (0.79)	16.0 (15.5)	0.69 (0.54)	9.0 (6.6)	73	
					**Change ETL to TNP‐Me**	0.82 (0.70)	16.1 (12.4)	0.68 (0.26)	9.0 (2.2)	25	
					**Change ETL to TNP‐H**	0.81 (0.76)	16.2 (15.2)	0.67 (0.40)	8.9 (4.6)	52	
PTB7‐Th:PC_71_BM	w/o	w/o	w/o	61 days	**w/o (PFN as ETL)**	0.81	16.2	0.68	8.9	54	^[^ ^201^ ^]^
					**Change ETL to FGr**	0.81	17.2	0.68	9.5	95	

**Table 5:** The photovoltaic parameters of devices in stability enhancement strategies based on hole transport materials design and selection

	Test conditions										
Active layer	Light	Heat	Air (relative humidity)	Duration	**Strategy**	*V*_oc_ [V]	*J*_sc_ [mA cm^−2^]	FF	PCE [%]	PCE/PCE_0_ [%]	Ref.
PTB7:PC_71_BM	w/o	Room temp	w (na)	8 h	**w/o (PEDOT:PSS as HTL)**	0.73	14.8	0.66	7.1	10	^[^ ^202^ ^]^
		.			**Change HTL to S‐MoO_x_**	0.72	14.9	0.68	7.3	80	
P3HT:PCBM	w/o	Room temp	w (na)	80 days	**w/o (PEDOT:PSS as HTL)**	0.60	9.5	0.57	3.3	38	^[^ ^203^ ^]^
		.			**Change HTL to s‐MoO_x_**	0.58	10.3	0.51	3.0	80	
PCDTBT:PC_70_BM	w	na	w (na)	4 h	**w/o (PEDOT:PSS as HTL)**	0.81	7.2	0.51	3.0	88	^[^ ^204^ ^]^
					**Change HTL to MoO_3_**	0.83	7.2	0.56	3.4	95	
PTB7:PC_71_BM	w/o	Room temp	w (na)	20 h	**w/o (PEDOT:PSS as HTL)**	0.77	12.3	0.68	6.4	0	^[^ ^281^ ^]^
		.			**Change HTL to MoO3 NP**	0.75	13.0	0.58	5.7	80	
PTB7‐Th:PC_71_BM	w/o	na	w (na)	30 days	**w/o (PEDOT:PSS as HTL)**	0.80 (0.80)	14.7 (10.2)	0.68 (0.34)	8.1 (2.8)	35	^[^ ^205^ ^]^
					**Change HTL to nw‐WO_2.72_**	0.80 (0.80)	15.2 (12.2)	0.68 (0.62)	8.2 (6.0)	73	
PBDTTT‐ET:IEICO	w/o	Room temp	w/o	16 days	**w/o (PEDOT:PSS as HTL)**	0.82	16.5	0.65	8.8	70	^[^ ^206^ ^]^
		.			**Change HTL to NiO_x_**	0.81	17.5	0.64	9.1	85	
P3HT:PCBM	na	Room temp	w (40%)	11 days	**w/o (PEDOT:PSS as HTL)**	0.60	9.1	0.66	3.6	0	^[^ ^282^ ^]^
		.			**Change HTL to Nanocomposite (Iron oxide/hydroxide)**	0.60	9.6	0.67	3.8	50	
PTB7‐Th:PC_71_BM	na	Room temp	W (30%)	47 days	**w/o (PEDOT:PSS as HTL)**	0.74	16.5	0.63	7.6	0	^[^ ^207^ ^]^
		.			**Change HTL to O‐MoS_2_ QDs**	0.78	16.7	0.59	7.7	64	
P3HT:PCBM	ISOS‐D‐1			346 h	**w/o (PEDOT:PSS as HTL)**	0.53	10.6	0.68	3.8	0	^[^ ^208^ ^]^
					**Change HTL to P3HT‐Si(OEt)_3_ layer**	0.49	10.3	0.61	3.1	36	
P3HT:PCBM	w/o	Room temp	w (na)	15 days	**w/o (PEDOT:PSS as HTL)**	na	na	na	na	0	^[^ ^200^ ^]^
					**Change HTL to NiS_2.0_**	0.47	10.1	0.47	2.3	15	
PTB7‐Th:PC_71_BM	w/o	na	w/o	360 h	**w/o (PEDOT:PSS as HTL)**	0.79 (0.79)	16.2 (10.5)	0.68 (0.57)	8.8 (4.7)	53	^[^ ^210^ ^]^
					**Change HTL to BiOCl NPs**	0.79 (0.79)	18.4 (16.4)	0.68 (0.62)	9.9 (8.0)	80	

**Table 6:** The photovoltaic parameters of devices in stability enhancement strategies based on active layer modification

	Test conditions										
Active layer	Light	Heat	Air (relative humidity)	Duration	**Strategy**	*V*_oc_ [V]	*J*_sc_ [mA cm^−2^]	FF	PCE [%]	PCE/PCE_0_ [%]	Ref.
PBDB‐T:ITIC	w/o	na	w (na)	1000	**w/o**	0.92	16.1	0.68	10.0	74	^[^ ^213^ ^]^
				h	**Ternary strategy: add acceptor N2200**	0.93	16.9	0.73	11.4	80	
PTB7‐Th:PC_71_BM	w/o	100 ^°^C	w/o	1100 h	**w/o**	0.81	15.2	0.70	8.6	70	^[^ ^214^ ^]^
					**Ternary strategy: add acceptor N2200**	0.79	15.1	0.64	7.7	80	
PBDB‐T‐2F:IT‐4F	w	na	w (na)	72 h	**w/o**	0.84	20.9	0.70	12.3	61	^[^ ^215^ ^]^
					**Ternary strategy: add acceptor N2200**	0.83	19.5	0.74	11.9	86	
PBDB‐T:ITIC‐M	w	na	w (na)	240 h	**w/o**	0.91 (0.88)	17.2 (13.0)	0.65 (0.39)	10.2 (4.4)	43	
					**Ternary: add acceptor N2200**	0.90 (0.87)	14.8 (13.7)	0.70 (0.58)	9.3 (6.9)	74	
PTB7‐Th:PC_70_BM	na	60 ^°^C	w (23%)	180 min	**w/o**	0.77	18.7	0.61	8.9	52	^[^ ^216^ ^]^
					**Ternary strategy: add acceptor PTN**	0.78	21.5	0.68	11.4	72	
	na	80 ^°^C	w (23%)	180 min	**w/o**	0.77	18.7	0.61	8.9	52	
					**Ternary strategy: add acceptor PTN**	0.78	21.5	0.68	11.4	75	
	w	na	w (23%)	180 min	**w/o**	0.77	18.7	0.61	8.9	38	
					**Ternary strategy: add acceptor PTN**	0.78	21.5	0.68	11.4	68	
	w/o	Room temp.	w (23%)	55 days	**w/o**	0.77	18.7	0.61	8.9	41	
					**Ternary strategy: add acceptor PTN**	0.78	21.5	0.68	11.4	59	
PTB7‐Th:PC_70_BM	w/o	60 ^°^C	w/o	7 days	**w/o**	0.76	14.3	0.48	5.2	57	^[^ ^84^ ^]^
					**Ternary strategy: add acceptor ITIC‐Th**	0.81	20.0	0.57	8.9	66	
p‐DTS‐(FBTTh_2_)_2_:PC_71_BM	w/o	90 ^°^C	na	100 h	**w/o**	0.78	14.9	0.64	7.4	52	^[^ ^217^ ^]^
					**Ternary strategy: add acceptor NCBA**	0.79	17.1	0.68	9.2	80	
PBDB‐TF:PC_71_BM	w/o	130 ^°^C	na	12 h	**w/o**	0.95	12.2	0.58	6.8	40	^[^ ^218^ ^]^
					**Ternary strategy: add acceptor HC‐PCIC**	0.89	19.3	0.70	12.4	80	
PBDB‐T:ITIC	w	na	na	120 min	**w/o**	0.84	14.1	0.68	8.1	80	^[^ ^219^ ^]^
					**Ternary strategy: add acceptor PC_70_BM**	0.84	14.1	0.71	8.3	90	
PBT1‐C:IT‐2F	w/o	w/o	w (na)	60 days	**w/o**	0.88	17.3	0.73	11.0	92	^[^ ^220^ ^]^
					**Ternary strategy: add acceptor PC_71_BM**	0.89	18.2	0.75	12.2	96	
PTB7‐Th:IEICO‐4F	w/o	Room temp.	w/o	90 days	**w/o**	0.71 (0.66)	23.8 (22.7)	0.65 (0.47)	10.9 (7.0)	64	^[^ ^221^ ^]^
					**Ternary Strategy: add acceptor PC_71_BM**	0.69	25.1	0.70	12.0	90	
PTB7‐Th:ITIC	na	60 ^°^C	na	180 min	**w/o**	0.80	14.9	0.62	7.4	50	^[^ ^222^ ^]^
					**Ternary strategy: add donor C7**	0.81	18.4	0.68	10.0	80	
	na	80 ^°^C	na	180 min	**w/o**	0.80	14.9	0.62	7.4	38	
					**Ternary strategy: add donor C7**	0.81	18.4	0.68	10.0	75	
	na	100 ^°^C	na	180 min	**w/o**	0.80	14.9	0.62	7.4	42	
					**Ternary strategy: add donor C7**	0.81	18.4	0.68	10.0	80	
	w	na	na	150 min	**w/o**	0.80	14.9	0.62	7.4	38	
					**Ternary strategy: add donor C7**	0.81	18.4	0.68	10.0	75	
PTB7‐Th:PC_71_BM	w/o	80 ^°^C	na	180 min	**w/o**	0.78	17.5	0.65	9.1	37	^[^ ^223^ ^]^
					**Ternary strategy: add donor PyPI**	0.77	19.3	0.68	10.4	89	
	w	na	w (na)	180 min	**w/o**	0.78	17.5	0.65	9.1	41	
					**Ternary strategy: add donor PyPI**	0.77	19.3	0.68	10.4	73	
J61:m‐ITIC	w/o	70 ^°^C	w (na)	20 h	**w/o**	0.90	19.2	0.65	11.2	70	^[^ ^224^ ^]^
					**Ternary strategy: add acceptor ITIC‐Th**	0.91	19.9	0.70	12.6	80	
	W	na	w (na)	160 h	**w/o**	0.90	19.2	0.65	11.2	70	
					**Ternary strategy: add acceptor ITIC‐Th**	0.91	19.9	0.70	12.6	85	
BDTRh:PCBM	w/o	80 ^°^C	w/o	100 h	**w/o**	0.88	5.2	0.52	2.3	70	^[^ ^83^ ^]^
					**Single component strategy: change active layer to BDTRh‐PCBM**	0.97	7.0	0.36	2.4	100	
PBDB‐T‐Cl:PBI	w	na	na	300 h	**w/o**	na	na	na	na	70	^[^ ^229^ ^]^
					**Single component strategy: change active layer to PBDBPBI‐Cl**	0.90	10.8	0.65	6.3	95	
PCDTBT:PC_70_BM	w/o	80 ^°^C	na	10 days	**w/o**	0.85 (0.72)	9.1 (8.6)	0.50 (0.38)	3.7 (2.4	65	^[^ ^230^ ^]^
					**Bilayer strategy: change active layer to PCDTBT/PC_70_BM**	0.80 (0.81)	9.9 (8.6)	0.60 (0.59)	4.5 (4.6)	100	
PCPDTBT:PCBM	na	80 ^°^C	na	12 days	**w/o**	0.62	11.9	0.53	3.9	40	^[^ ^231^ ^]^
					**Bilayer strategy: change active layer to PCPDTBT/PCBM**	0.63	10.7	0.50	3.4	70	
PDCBT:PCBM:ITIC	na	100 ^°^C	na	100 h	**w/o**	0.92	11.5	0.43	4.5	28	^[^ ^232^ ^]^
					**Bilayer strategy: change active layer to PDCBT/ITIC:PCBM**	0.87	10.1	0.71	6.0	50	

**Table 7:** The photovoltaic parameters of devices in stability enhancement strategies based on carrier transport layer modification

	Test conditions										
Active layer	Light	Heat	Air (relative humidity)	Duration	**Strategy**	*V*_oc_ [V]	*J*_sc_ [mA cm^−2^]	FF	PCE [%]	PCE/PCE_0_ [%]	Ref.
P3HT:PC_61_BM	w	80 C	na	28 h	**w/o (ZnO as ETL)**	0.61	8.9	0.62	3.4	85	^[^ ^283^ ^]^
					**Doping and mixing strategy: change ETL to ZnO:PEI**	0.61	9.2	0.64	3.4	90	
p‐DTS‐(FBTTh_2_)_2_:PC_70_BM	w	na	w (na)	28 h	**w/o (ZnO as ETL)**	0.77	15.4	0.67	7.7	50	^[^ ^235^ ^]^
					**Doping and mixing strategy: change ETL to L‐ZnO**	0.78	17.3	0.68	9.2	90	
PTB7‐Th:PC_71_BM	w/o	Room temp.	w (na)	15 days	**w/o (ZnO as ETL)**	0.69	15.6	0.59	6.4	10	^[^ ^236^ ^]^
					**Doping and mixing strategy: change ETL to Al doped ZnO**	0.80	16.9	0.75	10.1	80	
PTB7:PC_71_BM	w	Room temp.	w (na)	5 h	**w/o (ZnO as ETL)**	0.68	18.2	0.47	5.8	40	^[^ ^237^ ^]^
					**Doping and mixing strategy: change ETL to Al doped ZnO**	0.65	18.9	0.49	6.1	80	
PBDB‐TF:IT‐4F	w/o	w/o	w/o	30 days	**w/o (ZnO as ETL)**	0.85 (0.58)	20.1 (18.6)	0.73 (0.43)	12.6 (4.6)	37	^[^ ^284^ ^]^
					**Doping and mixing strategy: change ETL to ZnO:PFB‐Br**	0.87 (0.87)	20.2 (20.2)	0.79 (0.78)	13.8 (13.7)	99	
PBDB‐T:ITIC	w	na	na	900 min	**w/o (ZnO as ETL)**	0.92	16.8	0.70	10.8	0	^[^ ^285^ ^]^
					**Doping and mixing strategy: change ETL to ZnO:PEOz**	0.94	17.8	0.74	12.4	50	
PTB7‐Th:IT‐4F	w/o	Room temp.	w/o	1000 h	**w/o (ZnO as ETL)**	0.79	15.1	0.63	7.1	60	^[^ ^286^ ^]^
					**Doping and mixing strategy: change ETL to ZnO:Li**	0.83	16.1	0.67	8.7	81	
P3HT:ICBA	w/o	Room temp.	w/o	500 h	**w/o (ZnO as ETL)**	0.59	9.5	0.56	3.2	60	^[^ ^287^ ^]^
					**Doping and mixing strategy: change ETL to ZnO:ZnWO4**	0.63	10.5	0.61	4.0	95	
P3HT:PC_60_BM	w	Room temp.	w/o	280 h	**w/o (ZnO as ETL)**	0.67	6.3	0.57	2.4	40	^[^ ^161^ ^]^
					**Doping and mixing strategy: change ETL to ZnO:PEI**	0.67	6.4	0.59	2.6	90	
PTB7‐Th:PC_71_BM	w	Room temp.	w (40%)	150 h	**w/o (PEDOT:PSS as HTL)**	0.76	14.1	0.73	7.8	10	^[^ ^163^ ^]^
					**Doping and mixing strategy: change HTL to PFT‐D doped PEDOT:PSS**	0.76	14.7	0.72	8.0	55	
PCDTBT:PC_71_BM	w/o	na	w (na)	180 h	**w/o (PEDOT:PSS as HTL)**	na	na	na	na	68	^[^ ^147^ ^]^
					**Doping and mixing strategy: change HTL to V2O5 doped PEDOT:PSS**	na	na	na	na	93	
PTB7:PC_71_BM	w/o	Room temp.	w/o	180 days	**w/o (ZnO as ETL)**	0.74	14.3	0.67	7.1	78	^[^ ^242^ ^]^
					**Bilayer strategy: change ETL to ZnO/Al**	0.75	15.2	0.70	8.0	85	
PTB7:PC_71_BM	na	Room temp.	w (na)	30 days	**w/o (PFN as ETL)**	0.74	13.0	0.64	6.2	50	^[^ ^238^ ^]^
					**Bilayer strategy: change ETL to M‐ITR‐GO/PFN**	0.76	15.1	0.70	7.2	85	
PTB7:PC_71_BM	w	Room temp.	w (na)	10 min	**w/o (ZnO as ETL)**	0.72 (0.68)	17.0 (14.1)	0.53 (0.48)	6.5 (4.7)	64	^[^ ^168^ ^]^
					**Bilayer strategy: change ETL to Csl/ZnO**	0.73 (0.71)	17.6 (15.4)	0.57 (0.53)	7.3 (5.8)	79	
P3HT:IC_60_BA	w/o	Room temp.	w/o	1500 h	**w/o (ZnO as ETL)**	0.78	10.0	0.65	5.0	0	^[^ ^162^ ^] ^
					**Bilayer strategy: change ETL to ZnO/HfO_2_**	0.81	10.8	0.72	6.3	60	
PTB7‐Th:PCBM	w/o	w/o	w/o	50 days	**w/o (PDIN as ETL)**	0.81	16.5	0.66	8.6	78	^[^ ^239^ ^]^
					**Bilayer strategy: change ETL to PDIN/BCP**	0.82	17.6	0.70	9.9	98	
PTB7:PC_71_BM	w/o	Room temp.	w/o	500 h	**w/o (TiO_2_ as ETL)**	0.71	14.8	0.62	6.5	35	^[^ ^240^ ^]^
					**Bilayer strategy: change ETL to TiO_2_/TPPZn**	0.72	14.9	0.66	7.1	50	
					**Bilayer strategy: change ETL to TiO_2_/TPPCOOHZn**	0.73	15.7	0.67	7.7	60	
					**Bilayer strategy: change ETL to TiO_2_/TPPtriazinegly_2_Zn**	0.75	17.1	0.68	8.7	80	
PTB7:PC_71_BM	w	w	w (na)	1000 h	**w/o (ZnO as ETL)**	0.73	15.4	0.68	7.8	82	^[^ ^146^ ^]^
					**Bilayer strategy: change ETL to ZnO/PO‐PhQNO**	0.75	16.3	0.70	8.5	90	
					**Bilayer strategy: change ETL to ZnO/PO‐PhQ**	0.75	16.4	0.73	9.0	90	
PTB7:PC_71_BM	w/o	RT	w (na)	1008 h	**w/o (ZnO as ETL)**	0.75	15.3	0.65	7.6	81	^[^ ^288^ ^]^
					**Bilayer strategy: change ETL to ZnO/PTDPQ**	0.76	16.1	0.71	8.7	88	
PTB7‐Th:PC_71_BM	w/o	na	w (na)	42 days	**w/o (SnO_2_ as ETL)**	0.69	16.7	0.48	5.5 (4.9)	90	^[^ ^289^ ^]^
					**Bilayer strategy: change ETL to SnO_2_/K_2_CO_3_**	0.77	16.9	0.53	6.9 (6.2)	90	
					**Bilayer strategy: change ETL to SnO_2_/Li_2_CO_3_**	0.77	16.9	0.51	6.7 (6.1)	90	
					**Bilayer strategy: change ETL to SnO_2_/Rb_2_CO_3_**	0.78	16.9	0.56	7.4 (6.9)	94	
P3HT:PCBM	na	na	w (na)	15 days	**w/o (PEDOT:PSS as HTL)**	0.46	13.4	0.63	4.0	0	^[^ ^243^ ^]^
					**Bilayer strategy: change HTL to GO/PEDOT:PSS**	0.49	15.4	0.64	4.8	30	
P3HT:PCBM	w	na	na	1 h	**w/o (PEDOT:PSS as HTL)**	0.55	7.4	0.62	2.5	78	^[^ ^244^ ^]^
					**Bilayer strategy: change HTL to PEDOT:PSS/PEG**	0.57	8.0	0.68	3.1	92	

**Table 8:** The photovoltaic parameters of devices in stability enhancement strategies based on electrode modification

	Test conditions										
Active layer	Light	Heat	Air (relative humidity)	Duration	**Strategy**	*V*_oc_ [V]	*J*_sc_ [mA cm^−2^]	FF	PCE [%]	PCE/PCE_0_ [%]	Ref.
PTB7‐Th:PC_71_BM	w/o	Room	w/o	24 h	**w/o (Al as electrode)**	0.76	16.3	0.70	8.7	75	^[^ ^290^ ^]^
					**Change electrode to Au**	0.76	15.8	0.71	8.6	99	
PTB7:PC_71_BM	w/o	Room temp.	w (na)	170 h	**w/o (Al as electrode)**	0.75	14.6	0.65	7.1	75	^[^ ^291^ ^]^
					**Change electrode to Ag**	0.75	14.8	0.72	8.0	96	
PTB7‐Th:PC_71_BM	w/o	Room temp.	w/o	30 days	**w/o (Cu grid as electrode)**	0.77	1.4	0.26	0.3	0	^[^ ^248^ ^]^
					**Change electrode to Cu grid/GR**	0.80	16.3	0.65	8.5	98	
	w/o	Room temp.	w (na)	170 h	**w/o (Cu grid as electrode)**	0.77	1.4	0.26	0.3	0	
					**Change electrode to Cu grid/GR**	0.80	16.3	0.65	8.5	40	
PBDB‐T:ITIC	w/o	na	w/o	10 days	**W/O (PH1000 as electrode)**	0.86	16.6	0.64	9.2	58	^[^ ^148^ ^]^
					**Change electrode to PEDOT:PSS:S‐AgNWs**	0.89	17.5	0.64	10.0	72	

**Table 9:** The photovoltaic parameters of devices in stability enhancement strategies based on an inverted device structure

	Test conditions										
Active layer	Light	Heat	Air (relative humidity)	Duration	**Strategy**	*V*_oc_ [V]	*J*_sc_ [mA cm^−2^]	FF	PCE [%]	PCE/PCE_0_ [%]	Ref.
PTB7‐Th:PC_71_BM	w/o	Room temp.	w (na)	48 h	**w/o**	na	na	na	na	30	^[^ ^256^ ^]^
					**Change to inverted device structure**	0.74	14.8	0.67	7.28	95	
PCDTBT:PC_71_BM	w/o	Room temp.	w (na)	10 h	**w/o**	0.88	9.1	0.57	4.6	10	^[^ ^292^ ^]^
					**Change to inverted device structure**	0.90	10.0	0.56	5.1	70	
P3HT:PC_61_BM	w/o	Room temp.	w (na)	48 h	**w/o**	0.64	9.1	0.67	3.9	0	^[^ ^257^ ^]^
					**Change to inverted device structure**	0.62	10.9	0.63	4.3	95	
PTB7‐Th:PC_71_BM	w/o	Room temp.	w/o	46 days	**w/o**	na	na	na	9.7	33	^[^ ^293^ ^]^
					**Change to inverted device structure**	0.79	17.0	0.72	9.7	84	
PBDB‐T:ITIC	w/o	Room temp.	w (na)	45 days	**w/o**	0.90	15.6	0.67	9.7	40	^[^ ^104^ ^]^
					**Change to inverted device structure**	0.90	16.1	0.68	9.8	94	

**Table 10:** The photovoltaic parameters of devices in stability enhancement strategies based on processing strategies

	Test conditions										
Active layer	Light	Heat	Air (relative humidity)	Duration	**Strategy**	*V*_oc_ [V]	*J*_sc_ [mA cm^−2^]	FF	PCE [%]	PCE/PCE_0_ [%]	Ref.
PTB7‐Th:PC71BM	na	130 °C	na	1 h	**w/o (DIO as additive)**	0.80 (0.78)	15.6 (9.9)	0.66 (0.50)	8.3 (3.8)	46	^[^ ^265^ ^]^
					**Change additive to BT**	0.81 (0.80)	16.5 (15.2)	0.70 (0.56)	9.3 (6.9)	74	
PTB7‐Th:PC_71_BM	w	25 °C	w (35%)	55 h	**w/o (DIO as additive)**	0.80	16.5	0.47	6.2	32	^[^ ^294^ ^]^
					**Change additive to BTPE‐Rn**	0.80	18.6	0.60	8.9	57	
PBDB‐TF:IT‐4F	w	na	w (na)	120 h	**w/o (DIO as additive)**	0.89	18.8	0.71	11.9	84	^[^ ^267^ ^]^
					**Change additive to SA‐1**	0.86	20.2	0.76	13.3	86	
PBDB‐TT5:ITIC	w/o	150 °C	w/o	500 min	**w/o (DIO as additive)**	0.86	14.3	0.65	7.9	79	^[^ ^268^ ^]^
					**Change additive to PPFS**	0.89	15.9	0.66	9.3	82	
	w/o	w/o	w/o	20 days	**w/o (DIO as additive)**	0.86	14.3	0.65	7.9	79	
					**Change additive to PPFS**	0.89	15.9	0.66	9.3	84	
PTB7‐Th:ITIC	w/o	60 °C	na	250 min	**w/o (DIO as additive)**	0.80	17.5	0.59	8.5	32	^[^ ^295^ ^]^
					**Change additive to CN**	0.80	18.7	0.62	9.4	52	
					**Change additive to DDO**	0.81	18.4	0.62	9.4	70	
					**Change additive to ODO**	0.81	19.2	0.64	10.1	60	
	w	na	na	1000 min	**w/o (DIO as additive)**	0.80	17.5	0.59	8.5	38	
					**Change additive to CN**	0.80	18.7	0.62	9.4	50	
					**Change additive to DDO**	0.81	18.4	0.62	9.4	62	
					**Change additive to ODO**	0.81	19.2	0.64	10.1	62	
P3HT:PCBM	w/o	150 °C	w/o	5 h	**w/o**	0.60 (0.45)	9.5 (2.5)	0.63 (0.49)	3.6 (0.5)	15	^[^ ^145^ ^]^
					**Add additive BPA2EODMA**	0.61 (0.60)	9.7 (6.5)	0.67 (0.54)	4.0 (2.1)	54	
P3HT:PCBM	na	120 °C	w/o	120 min	**w/o**	na	na	na	na	50	^[^ ^296^ ^]^
					**Add additive 2CP**	0.57	10.0	0.48	2.7	96	
					**Add additive CHN**	0.55	8.4	0.49	2.3	92	
PTB7‐Th:IEICO‐4F	na	150 °C	w/o	200 min	**w/o**	0.70	23.2	0.67	10.8	60	^[^ ^297^ ^]^
					**Add additive BPNFs**	0.71	23.4	0.70	11.6	73	
PTB7:PC_71_BM	w/o	Room temp.	w (na)	350 h	**w/o (DIO as additive)**	0.76	16.0	0.68	8.0	83	^[^ ^269^ ^]^
					**Change additive to 4‐FBA/DIO co‐additives**	0.76	16.4	0.68	8.3	89	
PBDB‐T:ITIC					**w/o**	0.88	16.8	0.65	9.6	66	
					**Change additive to 4‐FBA/DIO co‐additives**	0.88	16.9	0.68	10.0	75	
PTB7‐Th:ITIC	ISOS‐D‐1			150 min	**w/o (o‐DCB as solvent)**	0.79	13.3	0.54	5.7	70	^[^ ^271^ ^]^
					**Change solvent to o‐MA**	0.83	14.7	0.46	5.6	78	
P19:ITIC	w/o	Room temp.	w (na)	750 h	**w/o (CB as solvent)**	0.94	15.2	0.70	10.0 (7.6)	76	^[^ ^272^ ^]^
					**Change solvent toluene**	0.94	15.7	0.68	10.0 (7.8)	78	
PBDB‐T:ITIC	w	Room temp.	w (na)	5 h	**w/o**	0.91 (0.84)	18.3 (14.5)	0.69 (0.45)	11.5 (5.5)	48	^[^ ^117^ ^]^
					**Thermal annealing**	0.91 (0.83)	18.3 (16.0)	0.67 (0.50)	11.1 (6.7)	60	
SAM‐72:PC71BM	w	na	w (na)	120 h	**w/o**	0.74	4.1	0.61	1.9	32	^[^ ^274^ ^]^
					**Thermal annealing + SVA annealing**	0.82	4.3	0.60	1.9	48	
F12TBT:PC_70_BM	w	Room temp.	w/o	3500 min	**w/o**	0.99 (0.96)	6.5 (5.3)	0.54 (0.52)	3.5 (2.7)	77	^[^ ^298^ ^]^
					**Thermal annealing at 70 °C**	1.00 (0.98)	6.7 (6.5)	0.52 (0.51)	3.5 (3.2)	92	
					**Thermal annealing at 100 °C**	1.01 (0.99)	6.5 (6.3)	0.50 (0.48)	3.3 (3.0)	92	
					**Thermal annealing at 140 °C**	1.01 (0.97)	5.1 (4.8)	0.41 (0.38)	2.1 (1.8)	85	
PTB7‐Th:PCDTBT:PC_71_BM	na	na	w (na)	7 days	**w/o**	0.73	15.9	0.64	7.4	85	^[^ ^275^ ^]^
					**Use methanol treatment**	0.79	17.9	0.6758	9.6	95	

### 4.1 Materials Design and Selection

#### 4.1.1 Donor Materials

The molecular structure of donor materials can have a profound impact on the stability of OSCs.^[^ ^172^ ^]^ Rational design of donor materials such as tuning the crystallinity and rigidity, incorporating antioxidant groups, and inducing copolymerization were all discovered as valid strategies to enhance the stability of the device.^[^ ^27^, ^93^, ^172^, ^173^, ^174^, ^175^, ^176^ ^]^ In recent 3 years, many research interests are focused on the side chain engineering of donor materials from the stability perspective.

**Figure 10:**
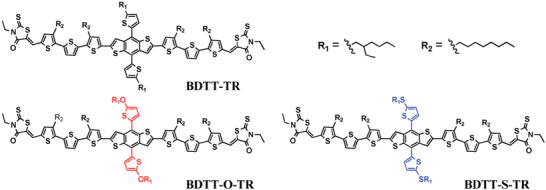
Chemical structures of BDTT‐TR, BDTT‐O‐TR, and BDTT‐S‐TR with different meta‐position bridging atoms in the side chain. Reproduced with permission.^[^ ^177^ ^]^ Copyright 2016, John Wiley and Sons.

**Figure 11:**
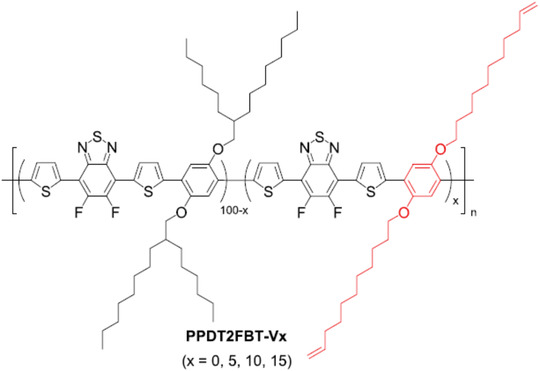
Chemical structures of PPDT2FBT‐V*_x_* with different contents of terminal vinyl‐appended side chains. Reproduced with permission.^[^ ^179^ ^]^ Copyright 2018, American Chemical Society.

**Figure 12:**
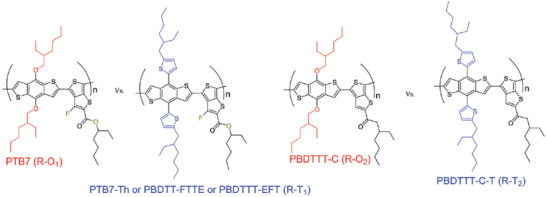
Chemical structures of PTB7‐Th and PBDTTT‐C‐T with alkylthienyl side chains and PTB7 and PBDTTT‐C with alkoxy side chains. Reproduced with permission.^[^ ^158^ ^]^ Copyright 2017, The Royal Society of Chemistry.

Brabec et al. found that small variations in the torsion angle of the side chains can cause the nature of the side chain linkage to be very different, which impacts on the thermal stability of the device.^[^ ^177^ ^]^ They designed and synthesized three donor materials bithienyl‐benzo[1,2‐b:4,5‐b′]dithiophene with meta‐alkyl side chain (BDTT‐TR), bithienyl‐benzo[1,2‐b:4,5‐b′]dithiophene with meta‐alkoxy side chain (BDTT‐O‐TR) and bithienyl‐benzo[1,2‐b:4,5‐b′]dithiophene with alkylthio side chain (BDTT‐S‐TR) with different meta‐position bridging atom in the side chain (as shown in **Figure** **10**) and blended these donors with fullerene acceptor PC_70_BM to fabricate devices. As results, the BDTT‐S‐TR based devices showed the best performance and thermal stability among the three. The BDTT‐S‐TR based devices maintained 35% of its initial PCE after annealed at 120 °C for 80 h which improved a lot when compared to the other two types of devices. Morgado et al. revealed that cross‐linking groups at the end of the side chain of donor materials could enhance the stability of the device. They modified donor oxetane‐functionalized polyfluorene‐alt‐bithiophene (F8T2Ox1) with oxetane moieties as cross‐linking groups in the side chain and paired it with PC_61_BM to fabricate devices. They tested the storage stability of both cross‐linked and non‐cross‐linked F8T2Ox1 based devices in an inert atmosphere for 24 days and found that the crosslk‐F8T2Ox1 based devices showed enhanced stability.^[^ ^178^ ^]^ Li and co‐worker proposed a similar strategy that adding UV cross‐linking in the side chain of donor materials can enhance the stability of the device.^[^ ^179^ ^]^ They designed and synthesized donor materials poly[(2,5‐bis(2‐hexyldecyloxy)phenylene)‐alt‐(5,6‐difluoro‐4,7‐di(thiophen‐2‐yl)benzo[c]‐[1,2,5]thiadiazole)] (PPDT2FBT)‐V*_x_* with different contents of terminal vinyl‐appended side chains for cross‐linking (as shown in **Figure** **11**) and found that the PPDB2FBT‐V_10_ based devices showed much higher thermal stability than PPDB2FBT based devices. The PPDB2FBT‐V_10_ based devices showed no PCE loss after being annealed at 120 °C for 40 h, while PPDB2FBT based devices degraded by over 40% of its initial PCE. Koster et al. recently proved that polymer donor with alkoxy side chains could cause higher stability in OSCs when compared to those polymer donors with alkylthienyl side chains.^[^ ^158^ ^]^ They compared widely used donors PTB7‐Th and poly[[4,8‐bis[5‐(2‐ethylhexyl)‐2‐thienyl]benzo[1,2‐b:4,5‐b′]dithiophene‐2,6‐diyl][2‐(2‐ethyl‐1‐oxohexyl)thieno[3,4‐b]thiophenediyl]] (PBDTTT‐C‐T) with their corresponded donors PTB7 and poly[(4,8‐bis‐(2‐ethylhexyloxy)‐benzo(1,2‐b:4,5‐b9)dithiophene)‐2,6‐diyl‐alt‐(4‐(2‐ethylhexanoyl)‐thieno[3,4‐b]thiophene‐)‐2‐6‐diyl)] (PBDTTT‐C) with alkoxy side chains (as shown in **Figure** **12**) and found that the PTB7 and PBDTTT‐C based devices showed higher light stabilities. Both PTB7 and PBDTTT‐C based devices can maintain over 80% of its initial PCE after being aged under UV light in inert atmosphere for 2 h, while PTB7‐Th and PBDTTT‐C‐T based devices degraded very fast under the same condition. They found that the fast decomposition of alkylthienyl side chain which creates free radical species and deep traps can be the root reason for the low stability. Polymer donor with alkylthienyl side chains is not promising for stable OSCs. More recently, they further demonstrated this conclusion by comparing donors poly[(4,8‐bis‐(2‐ethylhexyloxy)‐benzo(1,2‐b:4,5‐b′)dithiophene)‐2,6‐diyl‐alt‐((2‐ethylhexyl)‐thieno(3,4‐b)thiophene‐4‐carboxylate))‐2,6‐diyl)] (PBDTTT‐E) with alkoxy side chains and poly[(4,8‐bis(5‐(2‐ethylhexyl)thiophen‐2‐yl)‐benzo[1,2‐b;4,5‐b]dithiophene)‐2,6‐diyl‐alt‐(4‐(2‐ethylhexyl)‐thieno[3,4‐b]thiophene‐4‐carboxylate))‐2,6‐diyl)] (PBDTTT‐E‐T) with alkylthienyl side chains.^[^ ^73^ ^]^ As a result, PBDTTT‐E based devices showed only 18% of PCE loss after the UV degradation for 2 h in the inert atmosphere, while PBDTTT‐E‐T based devices showed 36% of PCE loss. By using proton nuclear magnetic resonance (H NMR) spectrum, they clearly demonstrated that the cleavage of alkylthienyl side chains which resulting in more electron traps is one of the root reasons for the UV instability in PBDTTT‐E‐T based devices.

**Figure 13:**
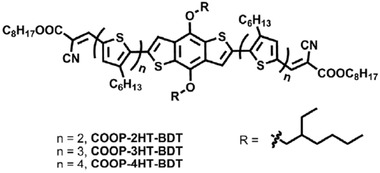
Chemical structures of small molecular donors COOP‐nHT‐BDT with different *π*‐conjugation chain length. Reproduced with permission.^[^ ^180^ ^]^ Copyright 2016, Elsevier.

**Figure 14:**
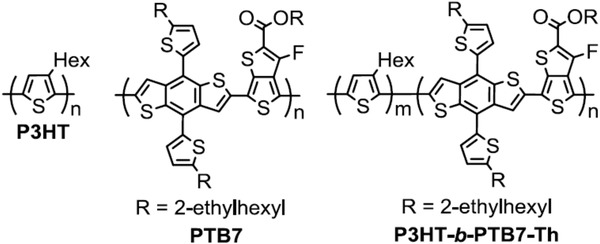
Chemical structures of polymer donor P3HT, PTB7, and copolymer P3HT‐b‐PTB7‐Th. Reproduced with permission.^[^ ^181^ ^]^ Copyright 2018, Elsevier.

Besides side chain engineering, some other molecular design strategies of donor materials were also reported. Ma et al. designed a series of A‐*π*‐D‐*π*‐A type conjugated small molecular donors benzo[1,2‐b:4,5‐b']dithiophene (BDT) electron‐donating core with 2‐cyano‐3‐octyloxy‐3‐oxo‐1‐propenyl (COOP) terminal electron‐accepting unit, and two regioregular oligo(3‐hexylthiophene) (nHT) (COOP‐nHT‐BDT) with different *π*‐conjugation chain length (as shown in **Figure** **13**) and paired these donors with fullerene acceptor PC_71_BM to fabricate devices.^[^ ^180^ ^]^ They found that the long‐term light stability of the device is positively related to the *π*‐conjugation chain length of the donor material. The COOP‐4HT‐BDT based device showed the highest stability among those devices, which maintained 85% of its initial PCE after being illuminated in an inert atmosphere for 320 h. Their work indicates the direction for the design of small molecular donor materials toward stable OSCs. On the other hand, Chen et al. reported the backbone engineering strategy of polymer donors.^[^ ^141^ ^]^ They compared donors PBDTTT‐C and PBDTTT‐C‐T with their corresponded donors PTB7 and PTB7‐Th with fluorine atoms in the backbone and found that the specimens containing fluorine atoms showed higher light stability when fabricated in OSCs. Both PTB7 and PTB7‐Th based devices maintained over 85% of its initial PCE after the burn‐in degradation for 1 h, while PBDTTT‐C and PBDTTT‐C‐T based devices degraded by over 30% of their device performance under the same condition. They pointed out that the higher domain purity contributed by fluorine atoms is the leading cause for high stability. Avgeropoulos and co‐workers also reported similar works that fluorine atoms in the backbone of polymer donor materials could reduce the microstructure instability in the device and increase the stability under the burn‐in condition.^[^ ^125^ ^]^ Recently, Horie et al. demonstrated a new strategy in polymer donor design toward stable OSCs.^[^ ^181^ ^]^ They designed all‐conjugated block copolymers P3HT‐b‐PTB7‐Th based on the two widely used donor P3HT and PTB7‐Th (as shown in **Figure** **14**). The P3HT‐b‐PTB7‐Th based devices showed impressive stability in the burn‐in test, which maintained 90% of its initial PCE after tested for 150 h. In comparison, both P3HT and PTB7‐Th based devices degraded by over 80% of their performance. The co‐polymer design is proved as a promising direction toward stable OSCs.

**Figure 15:**
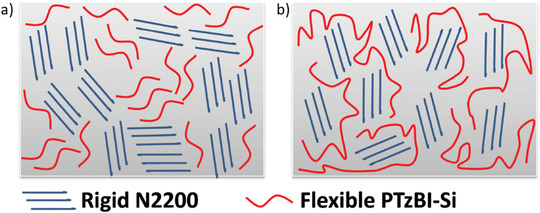
Aggregation tendencies and templating effects on the resulting morphologies of a) PTzBI‐Si_L_:N2200 and b) PTzBI‐Si_H_:N2200 based blend films. Reproduced with permission.^[^ ^182^ ^]^ Copyright 2019, Elsevier.

In addition to the molecular design of donor materials, Cao et al. revealed the relationship between the molecular weight and the stability of the device very recently.^[^ ^182^ ^]^ They synthesized two batches of polymer donor pyrrolo[3,4‐f]benzotriazole‐5,7‐dione (TzBI) building block with benzodithiophene electron‐rich unit (PTzBI‐Si) with high and low molecular weight, represented as PTzBI‐Si_H_ and PTzBI‐Si_L_. When paired with polymer acceptor poly{[N,N′‐bis(2‐octyldodecyl)‐naphthalene‐1,4,5,8‐bis(dicarboximide)‐2,6‐diyl]‐alt‐5,5′‐(2,2′‐bithiophene)} (N2200) in OSCs, the PTzBI‐Si_H_ based device showed both enhanced storage stability in the glove box and thermal stability under annealing at 80 °C when compared to PTzBI‐Si_L_ based devices. They found that the higher molecular weight of the donor can cause better miscibility between donor and acceptor in the active layer (as shown in **Figure** **15**). Their work emphasized the priority of using a polymer with a high molecular weight in the fabrication of all‐polymer solar cells.

Additionally, the rational design of donor materials can also increase the mechanical stability of OSCs. Lipomi et al. demonstrated that rationally decreasing the crystallinity of the polymer donor materials can increase the mechanical compliance of donor polymers without deleteriously affecting their optoelectronic properties.^[^ ^183^ ^]^ Moreover, Bao et al. pointed out that the increase of the flexibility of the polymer chain in the design of donor materials can be another way to increase the mechanical compliance of the device.^[^ ^184^ ^]^

#### 4.1.2 Acceptor Materials

In the past few years, non‐fullerene acceptors, owing advantages of easy tenable energy level, crystallinity, and absorption spectrum, have attracted many research interests.^[^ ^185^, ^186^ ^]^ The replacement of fullerenes with non‐fullerene acceptors were proved to effectively boost the PCE of OSCs over 16%.^[^ ^21^, ^22^, ^187^ ^]^ Meanwhile, it was also intensively studied from the stability perspective.^[^ ^188^, ^189^ ^]^

**Figure 16:**
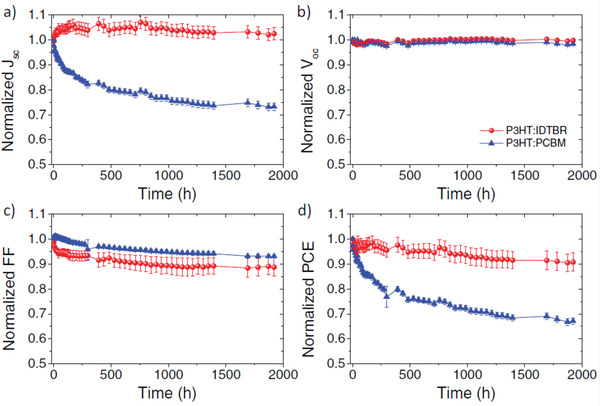
Normalized parameters of *J*_sc_, *V*_oc_, FF, and PCE of P3HT:IDTBR and P3HT:PCBM solar cells in the course of 2000 h of LED light exposure. Reproduced with permission.^[^ ^143^ ^]^ Copyright 2017, John Wiley and Sons.

**Figure 17:**
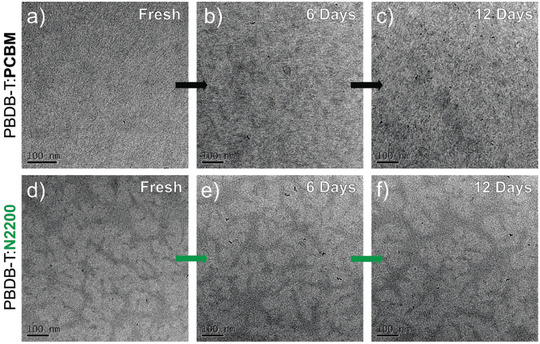
The comparison between PCBM and N2200 based active layer in morphology changing during the aging process a) and d) fresh, b) and e) 6 days and c) and f) 12 days in an inert atmosphere. Reproduced with permission.^[^ ^193^ ^]^ Copyright 2019, John Wiley and Sons.

In 2016, Jung et al. demonstrated the excellent thermal stability of non‐fullerene acceptor di‐PBI based devices.^[^ ^190^ ^]^ They compared the bay‐linked perylene bisimide derivative (di‐PBI) non‐fullerene based OSCs with its PC_71_BM fullerene counterpart and found that the replacement of fullerene with di‐PBI based non‐fullerene acceptor can significantly enhance the thermal stability of the device. The fabricated di‐PBI based non‐fullerene device can maintain over 70% of its initial PCE after heated at 100 °C for over 3 h, while the PC_71_BM based device degraded very fast in a few minutes. Similar strategies were also proved by Lee's group and Min's group. Lee et al. replaced fullerene acceptor PCBM with their newly designed non‐fullerene acceptor 3D‐shaped spirobifluorene and hexyl rhodamine (SF‐HR) in P3HT based OSCs and found that both thermal stability and burn‐in stability of the device showed a profound enhancement.^[^ ^191^ ^]^ Min et al. reported that both ITIC and 2,2ʹ‐((2Z,2ʹZ)‐((4,4,9,9‐tetrahexyl‐4,9‐dihydro‐s‐indaceno[1,2‐b:5,6‐bʹ]dithiophene‐2,7‐diyl)bis(methanylylidene))bis(3‐oxo‐2,3‐dihydro‐1H‐indene‐2,1‐diylidene))dimalononitrile (IDIC) based non‐fullerene OSCs exhibited better thermal stability than PC_70_BM based fullerene OSCs under the heat treatment at 100 °C.^[^ ^192^ ^]^ The better stability of non‐fullerene based OSCs in these studies was found mainly attributed to the reduced aggregation of acceptor during the aging process. From the perspective of light stability, the application of non‐fullerene acceptor IDTBR played a significant role in recent studies. Brabec and co‐workers reported an efficient burn‐in free device based on P3HT:IDTBR active layer in 2017.^[^ ^143^ ^]^ The fabricated IDTBR based devices showed excellent stability under the continuous illumination of the simulated Light emitting diode light, which maintained 97% of its initial PCE after 2000 h (as shown in **Figure** **16**). In comparison, the PC_70_BM based fullerene device can only maintain 67% of its initial PCE under the same condition. Later, Durrant's group and McCulloch's group reported similar works in the following 2 years. They paired EH‐IDTBR with donor PffBT4T‐2OD and PTB7‐Th to fabricate devices, respectively. As a result, both PffBT4T‐2OD:EH‐IDTBR and PTB7‐Th:EH‐IDTBR based devices showed profoundly enhanced light stability when compared to their fullerene based counterparts.^[^ ^142^, ^144^ ^]^ It was found that the replacement of fullerene acceptor with non‐fullerene acceptor IDTBR can both eliminate the fullerene dimerization and the increased energy disorder in the device during the burn‐in process.^[^ ^143^, ^189^ ^]^ In addition, the replacement of fullerene with polymer acceptor in OSCs was recently proved also useful to enhance stability. Ma et al. replaced PCBM with polymer acceptor N2200 in both PBDB‐T and PTzBI donor based active layer to fabricate devices.^[^ ^193^ ^]^ They found that the polymer acceptor N2200 based device showed excellent long‐term stability under an inert atmosphere with or without thermal stress and ambient condition without encapsulation. The N2200 polymer acceptor based active layer exhibited a clear and stable network morphology which rarely changes in the aging process, while PCBM based active layer showed aggregation (as shown in **Figure** **17**). They pointed out that the all‐polymer OSCs is another promising direction toward stable OSCs.

**Figure 18:**
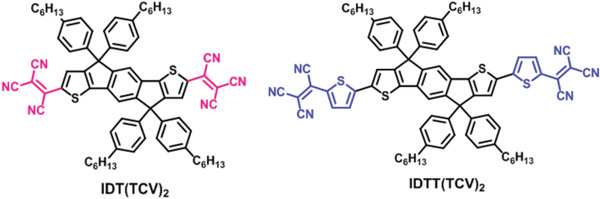
Chemical structure diagrams of small molecular non‐fullerene acceptors IDT(TCV)_2_ and IDTT(TCV)_2._ Reproduced with permission.^[^ ^194^ ^]^ Copyright 2017, American Chemical Society.

**Figure 19:**
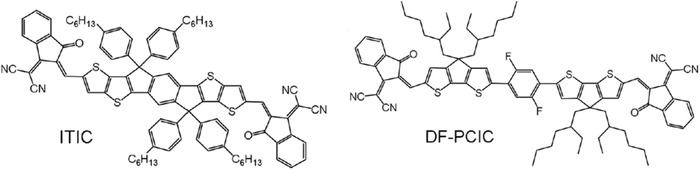
Chemical structure diagrams of small molecular non‐fullerene acceptors ITIC and DF‐PCIC. Reproduced with permission.^[^ ^195^ ^]^ Copyright 2017, John Wiley and Sons.

**Figure 20:**
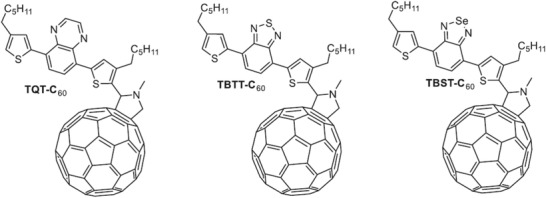
Chemical structure diagrams of modified novel fullerene acceptors TQT‐C_60_, TBTT‐C_60_, and TBST‐C_60_. Reproduced with permission.^[^ ^196^ ^]^ Copyright 2018, John Wiley and Sons.

Besides the replacement of fullerene acceptors, rational design of acceptor materials is another widely used strategy to enhance the stability of OSCs. Choi et al. designed and synthesized two small molecular acceptors IDT(TCV)_2_ and IDTT(TCV)_2_ based on tricyanovinylene (TCV) electron‐accepting unit and paired them with donor PTB7‐Th to fabricate devices (as shown in **Figure** **18**).^[^ ^194^ ^]^ They found both IDT(TCV)_2_ and IDTT(TCV)_2_ based devices showed superior shelf‐life stability that can be stable after being stored in air for 1000 h without any performance reduction. In comparison, the PC_70_BM based devices degraded by 40% of its performance under the same condition. They revealed that the excellent stability of these two non‐fullerenes based devices was ascribed to the rational tuned LUMO level of and the robust internal morphology induced by TCV‐containing acceptors. Chen et al. designed and synthesized a new non‐fullerene acceptor DF‐PCIC based on an unfused‐ring core containing two cyclopentadithiophene (CPDT) moieties and one 2,5‐difluorobenzene (DFB) group, as shown in **Figure** **19**.^[^ ^195^ ^]^ They compared DF‐PCIC with the state‐of‐the‐art non‐fullerene acceptor ITIC in the device from the stability perspective and found that the DF‐PCIC based devices showed better thermal stability maintaining 88% of its initial PCE after the heat treatment at 150 °C for 12 h. They suggested that the stable morphology induced by the unique unfused‐ring core of DF‐PCIC is the main reason. Moreover, in the rational design of acceptor materials, many studies focused on the modification of the well‐studied acceptors. Wang et al. linked three types of bulkier qulnoxaline (TQT), benzothiadiazole (TTBT), and benzoselenadiazole (TBST) to the fullerene acceptor and designed three modified novel fullerene acceptors TQT‐C_60_, TBTT‐C_60_, and TBST‐C_60_ (as shown in **Figure** **20**), respectively.^[^ ^196^ ^]^ When paired with donor P3HT, they found that all three types of modified fullerene based devices showed excellent thermal stability which all maintained over 90% of its initial PCE after being heated at 130 °C for 5 h. They proved that those added bulkier groups could act as an anchoring group to suppress the aggregation of fullerene acceptors in the active layer during the aging. Recently, Brabec et al. reported the excellent stability in ITIC derivatives based OSCs.^[^ ^28^ ^]^ Later, researcher also began to pay attention to the modification of ITIC from the stability perspective. Koster et al. compared the device photostability based on three ITIC derivatives ITIC, 3,9‐bis(2‐methylene‐((3‐(1,1‐dicyanomethylene)‐6/7‐methyl)‐indanone))‐5,5,11,11‐tetrakis(4‐hexylphenyl)‐dithieno[2,3‐d:2′,3′‐d′]‐s‐indaceno[1,2‐b:5,6‐b′]dithiophene, and 3,9‐bis(2‐methylene‐((3‐(1,1‐dicyanomethylene)‐6,7‐difluoro)‐indanone))‐5,5,11,11‐tetrakis(4‐hexylphenyl)‐dithieno[2,3‐d:2′,3′‐d′]‐s‐indaceno[1,2‐b:5,6‐b′]dithiophene.^[^ ^197^ ^]^ They found those three types of devices degraded differently under continuous illumination. The IT‐M based devices showed the best stability among three which maintained 88% of its initial PCE after aging for 2 h. They revealed that subtle changes in molecular structure coupled with imbalance charge mobilities might be the origin of the stability difference for different ITIC derivatives based devices. He et al. designed ITIC‐2Cl‐m derivatives by modifying the ITIC with adding chlorine atoms to different positions of chlorine atoms and synthesized two novel non‐fullerene acceptors ITIC‐2Cl‐*δ* and ITIC‐2Cl‐*γ*.^[^ ^198^ ^]^ When compared with ITIC‐2Cl‐m based devices, both ITIC‐2Cl‐*δ* and ITIC‐2Cl‐*γ* based devices showed enhanced light and thermal stability when under continuous illumination and heat treatment at 80 °C, respectively. They found that the different position of the chlorine actions in ITIC derivatives can have impact on the molecular orientation and packing in the active layer. The best stability of ITIC‐2Cl‐*γ* based devices is mainly attributed to its induced stable 3D interpenetrating network morphology in the active layer.

#### 4.1.3 Electron Transport Materials

Recently, there are also many newly designed materials used in electron transport layers toward stable OSCs. Xu et al. designed and synthesized fluorescent carbon quantum dots (C‐CQDs) to replace the lithium fluoride (LiF) as the electron transport layer in PTB7‐Th:PC_61_BM based OSCs.^[^ ^199^ ^]^ They proved that the device with C‐CQDs as the electron transport layer owns better thermal stability than the device with LiF as the electron transport layer, which maintained 82% of its initial PCE after being annealed at 80 °C for 160 h. The reduced diffusion possibility of electron transport materials in the C‐CQDs based electron transport layer is identified as the main reason for its better stability. Boujelbene et al. designed two novel hybrid compounds [C_5_H_8_N_3_]_2_[BiCl_5_] and [C_5_H_8_N_3_]_12_[BiCl_6_]_4_ referred as H1 and H2 to replace ZnO as the electron transport layer in the PTB7‐Th:PC_70_BM based OSCs.^[^ ^200^ ^]^ They pointed out that the ZnO based electron transport layer can inherently react with Al electrode forming an ultrathin aluminium oxide layer to degrade the device. With the replacement of H1 or H2 as the electron transport layer, the contact between the electron transport layer and the Al electrode become much stable. Both H1 and H2 based devices showed enhanced storage stability in the air when compared to ZnO based devices. Sun et al. synthesized a novel non‐conjugated polymer poly‐(sodium 2‐acrylamido‐2‐methylpropane sulfonate‐co‐styrene)/magnetite (PAMPS‐Na) to replace Ca as the electron transport layer in OSCs.^[^ ^166^ ^]^ They demonstrated that the PAMPS‐Na based devices showed superior storage stability than Ca based devices which maintained over 75% of its initial PCE after stored in air for 30 days. It is not hard to find that the replacement of unstable metal or metal oxide materials with more stable materials in the electron transport layer is an effective strategy to enhance the stability of OSCs.

On the other hand, Kim et al. revealed that rational molecular modification could also be an effective way to solve the instability caused by metal oxide based electron transport layer.^[^ ^60^ ^]^ They modified TNP with different AcAc derivatives and synthesized three novel electron transport materials TNP‐Ph, TNP‐Me, and TNP‐H. As results, all these three modified materials based devices showed better stability than TNP based devices, where the TNP‐Ph based devices showed the best stability. It maintained over 70% of its initial PCE after light soaking for 1000 h which was found attributed to its strong *π*–*π* interaction between phenyl rings of TNP‐Ph. In addition, graphene derivatives were found as promising electron transport materials for stable OSCs. Dong et al. first used a highly dispersed functionalized reduced graphene oxide (FGr) to replace poly [(9,9‐bis(3'‐(*N*,*N*‐dimethylamino)propyl)‐2,7‐fluorene)‐alt‐2,7‐(9,9‐dioctylfluorene)] (PFN) as the electron transport layer in the PTB7‐Th:PC_71_BM based OSCs.^[^ ^201^ ^]^ The storage stability of the device was found significantly enhanced with FGr, where the FGr based devices only dropped 7.4% of its initial PCE after being stored for 61 days in an inert atmosphere. They found that the FGr based electron transport layer owns a better resistance to moisture and oxidation when compared to PFN. FGr can act as a barrier to protect the active layer.

#### 4.1.4 Hole Transport Materials

As mentioned earlier, the widely used hole transport material PEDOT:PSS is unstable and can cause excessive instabilities when used in OSCs. Therefore, in recent years, many studies are focused on the replacement of PEDOT:PSS with alternative hole transport materials. Metal oxides such as MoO*_x_*, nickel oxide (NiO*_x_*), and tungsten oxide (WO*_x_*) were proved as a better option than PEDOT:PSS. Zhang et al. introduced a solution‐processable MoO*_x_* film (S‐MoO*_x_*) as an alternative hole transport layer in PTB7:PC_71_BM based OSCs and found that the S‐MoO*_x_* based device showed superior air stability than PEDOT:PSS based device.^[^ ^202^ ^]^ The S‐MoO*_x_* based device maintained 80% of its initial PCE after being stored in air for 8 h without encapsulation, while the PEDOT:PSS based device can only maintain 10% of its initial PCE under the same condition. Their group also reported a similar work based on the application of S‐MoO*_x_* in P3HT:PCBM based OSCs.^[^ ^203^ ^]^ Moons et al. demonstrated that the replacement of PEDOT:PSS with MoO_3_ as a hole transport layer in PCDTBT:PC_70_BM based devices can enhance the light stability of the device. ^[^ ^204^ ^]^ They found the *V*_oc_ loss in the device can be significantly reduced by using the MoO_3_ based hole transport layer. Besides MoO*_x_* derivatives, Sun et al. proved the application of WO_2.72_ nanowire (nw‐WO_2.72_) as the alternative hole transport layer in OSCs.^[^ ^205^ ^]^ They found the replacement of PEDOT:PSS with nw‐WO_2.72_ can also profoundly enhance the air stability of the device. Tan et al. reported similar work based on the application of NiO*_x_* based hole transport layer.^[^ ^206^ ^]^

In addition to metal oxides, some other alternative materials were also reported. Huang et al. applied the MoS_2_ quantum dots (QDs) as the hole transport layer to replace the PEDOT:PSS in the PTB7‐Th:PC_61_BM based device and proved the superior air stability of MoS_2_ based devices.^[^ ^207^ ^]^ Chambon et al. designed a novel material P3HT‐Si(OEt)_3_ to replace the PEDOT:PSS as the hole transport layer in the P3HT:PCBM based device and found enhanced storage stability under ISOS‐D1 test condition.^[^ ^208^ ^]^ Han et al. proved the replacement of PEDOT:PSS with NiS derivatives,^[^ ^209^ ^]^ and Guo et al. reported a similar work based on the bismuth oxychloride (BiOCl) nanoplates (NPs).^[^ ^210^ ^]^ It is easy to find that the replacement of hole transport layer PEDOT:PSS is an effective method to increase the stability of OSCs. However, it is worth to mention that most of those alternative materials to PEDOT:PSS is evaporated materials which are not suitable for the application in the large‐scale and roll‐to‐roll fabrication of OSCs. Herein, we recommend that developing solution‐processable alternative materials which is compatible with roll‐to‐roll fabrication to PEDOT:PSS is a more meaningful way toward stable OSCs.

### 4.2 Device Engineering

#### 4.2.1 Active Layer Modification

##### Strategy

The ternary strategy has received increasing research attention in recent years. Ternary blend, which contains three organic materials either donor:donor:acceptor (D_1_:D_2_:A) or donor:acceptor:acceptor (D:A_1_:A_2_) combination, usually can give a higher device performance than its binary blend counterpart.^[^ ^12^, ^14^, ^211^, ^212^ ^]^ From the stability perspective, the ternary strategy was also intensively studied. The ternary strategy was widely applied as an effective strategy to improve the device stability in recent 3 years.

**Figure 21:**
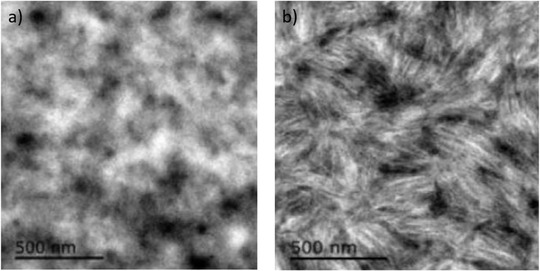
TEM images of a) PBDB‐T:ITIC binary blend and b) PBDB‐T:N2200:ITIC ternary blend. Reproduced with permission.^[^ ^213^ ^]^ Copyright 2017, Elsevier.

As mentioned, many recent studies found that the application of polymer acceptor materials in the binary blend can create a more stable morphology and enhance the stability of the device. Polymer acceptors were also recently found as a promising third component in the ternary blend to improve the stability. Zhang et al. added polymer acceptor N2200 as the third component into the PBDB‐T:ITIC non‐fullerene based binary system to fabricate ternary OSCs.^[^ ^213^ ^]^ It was found that the PBDB‐T:N2200:ITIC based ternary device not only showed an increased device performance but also exhibited enhanced air stability than the PBDB‐T:ITIC based binary device when stored in the air without encapsulations. Li et al. incorporated polymer acceptor N2200 into the PTB7‐Th:PC_71_BM fullerene based binary system as the third component.^[^ ^214^ ^]^ They proved that the addition of N2200 could also enhance the thermal stability of the device. The fabricated PTB7‐Th:N2200:PC_71_BM ternary device maintained 80% of its initial PCE after the heat treatment at 100 °C for 1100 h which enhanced by 10% when compared to PTB7‐Th:PC_71_BM based binary devices. Similarly, So et al. demonstrated the superior light stability of N2200 added ternary devices based on both PBDB‐T‐2F:IT‐4F and PBDB‐T:ITIC‐M based binary system.^[^ ^215^ ^]^ On the reviewing of those works, the origin of enhanced stability of the N2200 added ternary device is found attributed to the more stable morphology in the active layer. Polymer acceptor N2200 was found to act as a morphology stabilizer which can create a stable fibrillar structure in the blend film (as shown in **Figure** **21**).^[^ ^213^ ^]^ The more stable morphology of the polymer acceptor based ternary active layer is the leading cause contributed to the enhancement in the stability of the ternary device.

**Figure 22:**
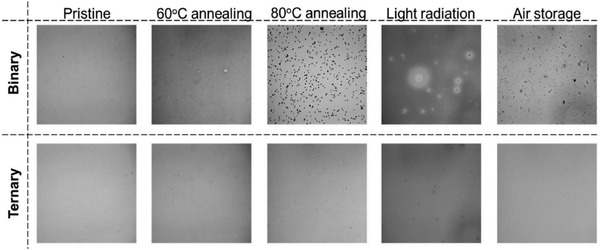
Optical microscopy images of PTB7‐Th:PC_71_BM based binary and PTB7‐Th:PTN:PC_71_BM based ternary films before and after the aging under annealing, light radiation, and air storage for 180 min. Reproduced with permission.^[^ ^216^ ^]^ Copyright 2018, The Royal Society of Chemistry.

Besides polymer acceptors, small molecular non‐fullerene and fullerene acceptor materials were also widely used as the third component in the ternary strategy. Many studies proved that the addition of another small molecular acceptor in the fullerene based binary system could enhance the stability of the device. Recently, Zhang et al. designed and synthesized a novel non‐fullerene acceptor material diphenylimidazole derivative (PTN) and added it into a PTB7‐Th:PC_71_BM based binary system to fabricate ternary OSCs. ^[^ ^216^ ^]^ As a result, they proved that the addition of PTN in the blend not only can increase the PCE but also can enhance the light, thermal, and air stability of the device. Based on the same binary system, Ko et al. did a similar study later.^[^ ^84^ ^]^ They added the non‐fullerene acceptor ITIC‐Th as the third component and proved an enhancement in the thermal stability of the device. Based on the 7,7′‐[4,4‐bis(2‐ethylhexyl)‐4H‐silolo[3,2‐b:4,5‐b′]dithiophene‐2,6‐diyl]bis[6‐fluoro‐4‐(5′‐hexyl‐[2,2′‐bithiophen]‐5‐yl)benzo[c][1,2,5]thiadiazole] (p‐DTS‐(FBTTh_2_)_2_):PC_71_BM based binary system, Wang et al. added a small molecular acceptor dihydronaphthyl based C60 bisadduct (NCBA) as the third component to fabricate ternary devices.^[^ ^217^ ^]^ They proved that the NCBA added ternary device could maintain 80% of its initial PCE after annealed at 90 °C for 100 h, while the binary device can only maintain 52% of its initial PCE under the same condition. Chen et al. observed the same effect when they added non‐fullerene acceptor 2,2′‐((2Z,2′Z)‐(((2,5‐difluoro‐1,4‐phenylene)bis(4,4‐bis(2‐ethylhexyl)‐4H‐cyclopenta[2,1‐b:3,4‐b′]dithiophene‐6,2‐diyl))bis(methanylylidene))bis(5,6‐difluoro‐3‐oxo‐2,3‐dihydro‐1H‐indene‐2,1‐diylidene))dimalononitrile (HC‐PCIC) into the PBDB‐TF:PC_71_BM based binary system.^[^ ^218^ ^]^ On the other hand, the addition of fullerene acceptor into a non‐fullerene based binary system was proved can enhance the stability of the device as well. Koster et al. added fullerene acceptor PC_71_BM into the PBDB‐T:ITIC based binary system and proved an enhancement in the light stability of the device.^[^ ^219^ ^]^ Sun et al. added PC_71_BM into the poly[[5,7‐bis(2‐ethylhexyl)‐4,8‐dioxo‐4H,8H‐benzo[1,2‐c:4,5‐cʹ]dithiophene‐1,3‐diyl][3,3ʹ''‐bis(2‐ethylhexyl)[2,2':5',2'':5'',2'''‐quaterthiophene]‐5,5'''‐diyl]] (PBT1‐C):IT‐2F based binary system and proved enhanced air stability.^[^ ^220^ ^]^ Similar works were also conducted by Ade et al. They added PC_71_BM into the PTB7‐Th:IEICO‐4F based binary system and found that the storage stability of the device can be profoundly improved in an inert atmosphere.^[^ ^221^ ^]^ Interestingly, it was found that the stability improvement in the ternary system with both non‐fullerene and fullerene acceptors is also attributed to its more stable morphology in the ternary blend. As shown in **Figure** **22**, in aging processes of PTN based ternary blend film, there are no noticeable morphology changes observed. However, the PTB7‐Th:PC_71_BM based binary blend without the addition of PTN showed huge changes with the occurring of large aggregation after the aging process.^[^ ^216^ ^]^ Ko et al. revealed that the ternary blends containing fullerene and non‐fullerene acceptors could benefit from the suppressed domain growth via kinetic and thermodynamic effects during the aging process and own a more stable morphology.^[^ ^84^ ^]^

In addition, some other ternary combinations such as D_1_:D_2_:A system and the system with two non‐fullerene acceptors were also recently reported owing the similar effect to enhance the stability of the device.^[^ ^222^, ^223^, ^224^ ^]^ Overall, it is easy to find that the morphology tuning in the active layer is the origin of the ternary strategy to enhance the stability of the device. To enhance the stability of the device by the ternary strategy, the addition of the third component must be capable of creating a more stable morphology in the active layer. Thus, rational selection of the third component is essential in the ternary strategy. It is suggested that the third component needs to have miscibility in the donor polymer and also needs to be partly miscible with the crystallizable acceptor to form a more stable morphology in the active layer.^[^ ^221^ ^]^

##### Single Component and Bilayer Strategy

As mentioned earlier, unstable morphology in the BHJ is one of the main barriers hindering the stability of the OSCs. Although various strategies have been developed to tune and improved the morphology, it is still hard to control and prevent the morphology evolution in the BHJ based active layer during the aging process.^[^ ^225^, ^226^, ^227^ ^]^ Therefore, many recent studies focused on the research of a single component which eliminates the unstable morphology problem.

Recently, Brabec et al. developed a new single component OSC based on a double‐cable conjugated polymer diketopyrrolopyrrole backbone with pendant perylene bisimide (SCP3).^[^ ^228^ ^]^ Impressively, they proved that the SCP3 based single component OSCs exhibited extremely high stability which showed no performance loss under the simultaneous one‐sun illumination and 90 °C heat treatment for 300 h. Woo et al. synthesized a novel donor–acceptor conjugate benzodithiophene‐rhodanine‐PCBM based on the BDTRH:PCBM based BHJ binary system to fabricate single component OSCs.^[^ ^83^ ^]^ As a result, the BDTRh‐PCBM based single component device showed excellent morphological and device stability which showed no performance degradation under the heat treatment at 80 °C for 100 h. In comparison, the BDTRh:PCBM based binary devices degraded by 30% of its performance under the same condition. Similarly, Li et al. designed a donor–acceptor conjugate PBDBPBI‐Cl based on the PBDB‐T‐Cl:perylene bisimide (PBI) based binary system on fabricating single component OSCs.^[^ ^229^ ^]^ The PBDBPBI‐Cl based device showed significantly enhanced light stability when compared to PBDB‐T‐Cl:PBI based binary device, which maintained 95% of its initial PCE after illuminated under one‐sun for 300 h. They found that this 5% performance loss in PBDBPBI‐Cl based device is due to the degradation in the interface layer which is unrelated to the active layer. Therefore, single component strategy can be considered as an effective method to eliminate the morphology degradation in the active layer and enhance the stability of OSCs.

Moreover, bilayer strategy for the active layer was also reported which can reduce morphology degradation and enhance the device stability in OSCs. By using the sequential deposition technique, donor and acceptor materials were deposited as separate layers to form the active layer. Bilayer structure can also prevent the unstable morphology problem, as there is no need to mix of donor and acceptor phase in the active layer. Kim et al. fabricated PCDTBT/PCBM based bilayer OSCs and compared it with the PCDTBT:PCBM based BHJ based OSCs.^[^ ^230^ ^]^ They proved that the bilayer devices could show both higher PCE and thermal stability than the BHJ device. The PCDTBT/PCBM based bilayer device showed no performance loss under the heat treatment at 80 °C for 10 days, while the PCDTBT:PCBM based BHJ based devices loss 35% of its initial PCE under the same condition. Similar work was also done based on the poly[2,6‐(4,4‐bis‐(2‐ethylhexyl)‐4H‐cyclopenta [2,1‐b;3,4‐b′]dithiophene)‐alt‐4,7(2,1,3‐benzothiadiazole)] (PCPDTBT):PC_71_BM based binary system.^[^ ^231^ ^]^ The bilayer device showed 30% of thermal stability enhancement compared to the BHJ device. More recently, Kim et al. found that the bilayer strategy is also valid on the ternary OSCs.^[^ ^232^ ^]^ They separately deposited the donor film and mixed acceptor film based on the poly[(4,4'‐bis(2‐butyloctoxycarbonyl‐[2,2'‐bithiophene]‐5,5‐diyl)‐alt‐(2,2'‐bithiophene‐5,5'‐diyl)] (PDCBT):ITIC:PCBM ternary system to fabricated devices. The PDCBT/ITIC:PCBM based bilayer device exhibited better thermal stability than the PDCBT:ITIC:PCBM BHJ device.

#### 4.2.2 Carrier Transport Layer Modification

##### Doping and Mixing

Based on widely applied carrier transport layers such as ZnO and PEDOT:PSS, doping and mixing strategy which incorporates another material into the carrier transport layer is one of the simplest methods without replacing materials to enhance the interfacial properties as well as the device performance in OSCs.^[^ ^233^, ^234^ ^]^Gratifyingly, recent studies found that many doping and mixing strategies were also useful for improving the stability of the device. For instance, Chand et al. doped sodium quantum dots into the ZnO electron transport layer in 7,7?‐[4,4‐Bis(2‐ethylhexyl)‐4H‐silolo[3,2‐b:4,5‐b?]dithiophene‐2,6‐diyl]bis[6‐fluoro‐4‐(5?‐hexyl‐[2,2?‐bithiophen]‐5‐yl)benzo[c][1,2,5]thiadiazole]:PC_70_BM based devices.^[^ ^235^ ^]^ The device with sodium quantum dots doped ZnO (L‐ZnO) not only showed increased PCE value but also exhibited superior light stability than the device with undoped ZnO. Fang's group and Uddin's group reported that doping Al into the ZnO could also enhanced both the performance and stability of the device.^[^ ^236^, ^237^ ^]^ Recently, Aziz et al. mixed ZnO with polyethylenimine (PEI) to fabricated electron transport layer in the P3HT:PC_60_BM based device.^[^ ^161^ ^]^ They found that the using of the mixed ZnO:PEI electron transport layer can significantly enhance the light stability of the device. The ZnO:PEI based device maintained 90% of its initial PCE after illuminated for 280 h, while the ZnO based device degraded by over 60% of its initial PCE under the same condition. Similar works were also reported on the PEDOT:PSS based hole transport layers. Moon et al. doped poly[9,9‐bis(4′‐sulfonatobutyl)fluorene‐alt‐thiophene] (PFT‐D) into the PEDOT:PSS hole transport layer and proved an enhancement in the device lifetime by five times in the ambient condition with 40% humidity.^[^ ^163^ ^]^ Sulaiman et al. incorporated vanadium oxide (V_2_O_5_) into the PEDOT:PSS based hole transport layer and found that the air stability of the device can be profoundly improved.^[^ ^147^ ^]^

On reviewing those works, there are two main origins found attributed to the stability enhancement by using the doping and mixing strategy in carrier transport layers. First, the doping or mixing carrier transport layers with suitable materials were found to form more stable contact interfaces. Doping Al into ZnO was found to remove unfavorable dipoles which can cause instability at the interface between the active layer and electron transport layer.^[^ ^236^ ^]^ The mixing of ZnO with PEI was proved to form a more electronically and energetically stable contact at the interface between the electrode and electron transport layer.^[^ ^161^ ^]^ Second, it was found that some doped or mixed carrier transport layers can act as a better blocker to resist exterior degradation factors such as air and water and internal degradation factors like the diffusion of the metal electrode atom. For example, the main reason for enhanced light stability in L‐ZnO based devices was that the L‐ZnO based electron transport layer can better protect the active layer from the UV light than the ZnO based electron transport layer.^[^ ^235^ ^]^ The Al doped ZnO was proved to own higher resistivity to the oxygen and water permeation.^[^ ^236^ ^]^ The V_2_O_5_ incorporated PEDOT:PSS was revealed to better hinder the diffusion of indium atoms from the anode.^[^ ^147^ ^]^

##### Bilayer Carrier Transport Layer Strategy

Bilayer strategy, which employed double carrier transport layers, was intensively reported as another effective method to enhance the device stability in the past 3 years. The origin of the bilayer strategy is similar to the doping and mixing strategy that an additional carrier transport layer can provide more stable interface contacts and act as the blocker for exterior and interior degradation factors in OSCs.^[^ ^168^, ^238^, ^239^, ^240^, ^241^ ^]^

**Figure 23:**
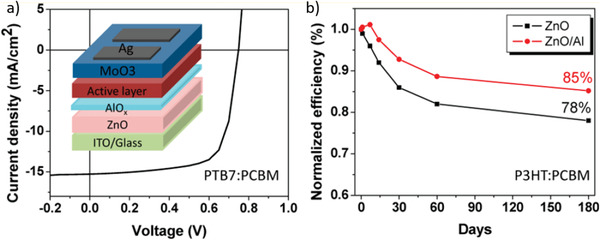
a) Device structure and IV curve of ZnO/Al bilayer based OSCs. b) Stability comparison between ZnO/Al bilayer based devices and reference devices. Reproduced with permission.^[^ ^242^ ^]^ Copyright 2017, American Chemical Society.

Hao et al. added an ultrathin Al layer on the top of the ZnO based electron transport layer to form a bilayer structure in PTB7:PC_71_BM based OSCs.^[^ ^242^ ^]^ It was found that the addition of Al layer can reduce the surface defects at the interface between the electron transport layer and the active layer and provide air resistance and oxygen scavenging capability. As a result, the device with the bilayer structured electron transport layer showed both enhanced device performance and shelf‐life stability, as shown in **Figure** **23**. More recently, Vasilopoulou et al. used the atomic layer deposition (ALD) to insert an ultrathin hafnium oxide (HfO_2_) layer between the ZnO layer and the active layer in the P3HT:indene‐C_60_bisadduct (IC_60_BA) based OSCs.^[^ ^162^ ^]^ The atomic deposited ultrathin HfO_2_ layer was proved to passivate the surface of the ZnO layer and form a more efficient and stable contact interface between the active layer and electron transport layer. As a result, they proved that the T_80_ lifetime of the device could be extended by three times by using the ZnO/HfO_2_ bilayer structure. On the other hand, some studies also applied bilayer strategy to modify the hole transport layer. Han et al. added a GO layer between the PEDOT:PSS layer and the electrode to form a bilayer structured hole transport layer.^[^ ^243^ ^]^ They found the additional graphene oxide (GO) layer can act as a blocker for the permeation of oxygen and moisture to the PEDOT:PSS layer and prevent the acidic PEDOT:PSS to damage the electrode. As a result, the device with GO/PEDOT:PSS bilayer based hole transport layer exhibited enhanced air stability. Similarly, Kishi et al. reported a PEDOT:PSS/PEG bilayer structured hole transport layer in the P3HT:PCBM based OSCs.^[^ ^244^ ^]^ Better light stability was proved by using the PEDOT:PSS/PEG bilayer in the device, as the additional poly(ethylene glycol) (PEG) layer was found suppressed the diffusion of PEDOT:PSS to the Al electrode during the light irradiation. Herein, although bilayer carrier transport layer strategy has been widely demonstrated as an effective method to improve the device stability, we think that it may not be a promising method from the perspective of the large‐scale fabrication of OSCs. Bilayer carrier transport layer strategy can increase the complexity of the device fabrication process and some materials such as ALD deposited on HfO_2_ is not even compatible with the roll‐to‐roll fabrication of OSCs. Thus, we recommend that the bilayer carrier transport layer strategy should be carefully used in future research.

#### 4.2.3 Electrode Modification

Some widely used electrodes in OSCs such as ITO and Al are unstable under exterior factors including irradiation, mechanical stress, and moistures and can cause instabilities in the device.^[^ ^27^, ^245^ ^]^ Thus, using more stable electrodes is a straightforward strategy to increase device stability. Replacing and modifying the electrode with more stable materials such as metal and alloy, graphene, polymer, and metal nanowires were intensively studied as valid methods toward stable OSCs.^[^ ^245^, ^246^, ^247^ ^]^ Chen et al. recently designed a novel PEDOT:PSS:S‐ silver nanowires (AgNWs) compound to replace Clevios Poly(3,4‐ethylenedioxythiophene)‐poly(styrenesulfonate) as the electrode in the PBDB‐T:ITIC based OSCs.^[^ ^148^ ^]^ They proved that the PEDOT:PSS:S‐AgNWs based electrode owned a higher PH value than PH1000 based electrode, which reduced the corrosive process in the electrode. As a result, the PEDOT:PSS:S‐AgNWs based device showed superior storage stability than the PH1000 based device which retain 72.1% of its original PCE value after storage for 10 days. More recently, Park and co‐workers designed and applied a novel copper (Cu) grid/graphene hybrid electrode in the PTB7‐Th:PC_71_BM based OSCs.^[^ ^248^ ^]^ It was found that the graphene can prevent the damage of Cu grid during the aging process. They proved that the Cu grid/graphene based device could exhibit better both inert and ambient storage stability.

#### 4.2.4 Inverted Device Structure

**Figure 24:**
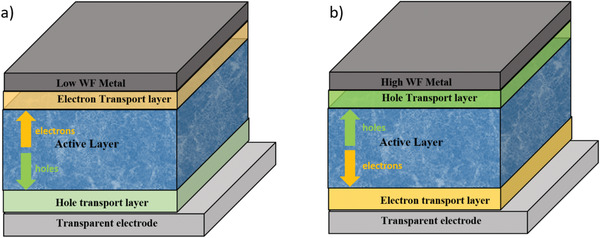
Schematic diagrams of a) conventional and b) inverted device structure for OSCs.

In OSCs, the active layer is always sandwiched between the low work function (WF) metal cathode such as (aluminium (Al), calcium (Ca), and barium (Ba)) and the high WF transparent conducting metal oxides anodes such as ITO and fluorine doped tin oxide (FTO).^[^ ^249^, ^250^ ^]^ To date, there are two types of widely reported device structures for OSCs which are called as conventional and inverted device structures, respectively.^[^ ^249^, ^250^, ^251^, ^252^, ^253^ ^]^ The schematic diagram of a typical conventional and inverted device structure has been shown in **Figure** **24**. The difference in the device structure can cause stability difference for OSCs.^[^ ^254^, ^255^ ^]^ Commonly, OSCs with conventional device structure own bad air stability.^[^ ^256^, ^257^, ^258^ ^]^ In the OSC with conventional device structure, the cathode with low WF is deposited at the exposed surface which can be easily damaged by exterior factors like oxygen and moistures.^[^ ^27^, ^30^ ^]^ The inverted device structure somehow can overcome this issue.^[^ ^259^, ^260^ ^]^ By using the inverted device structure, the position of the anode and the cathode are exchanged where the cathode is stacked underneath the exposed surface.^[^ ^261^, ^262^, ^263^ ^]^ Therefore, the inverted device structure can prevent the cathode from the permeation of oxygen and moisture and can cause better air stability in OSCs.^[^ ^27^, ^250^ ^]^ For example, Choulis et al. proved that OSCs with the inverted device structure of ITO/ZnO/P3HT/PC_61_BM/PEDOT:PSS/Ag can preserve 95% of its initial PCE value after exposing to the air for 1 h, while OSCs with the conventional device structure of ITO/PEDOT:PSS/P3HT/PC_61_BM/ZnO/Al can only preserve 20% of its initial PCE under the same condition.^[^ ^258^ ^]^ Similarly, Chang et al. found that OSCs based on PTB7/PC_71_BM system with the inverted device structure can exhibit much better air stability than the device with a conventional device structure. The fabricated PTB7/PC_71_BM based inverted device can maintain 95% of its original PCE value after exposing to air for 48 h, while the PTB7/PC_71_BM based conventional device can only maintain 30% of its original PCE value without device encapsulation.^[^ ^256^ ^]^

Nevertheless, it is worth to mention that the use of an inverted device structure is a limited strategy to improve device stability. Although the inverted device structure can effectively prevent air penetration. The inverted device may less stable than the conventional device under other degradation factors such as light and heat. McCehee et al. recently did a comparative study between the conventional and inverted device structure based on the PCDTBT:PC_71_BM system from the perspective of the long‐term operation.^[^ ^264^ ^]^ In their study, both types of as‐fabricated devices were exposed to continuous one‐sun illumination in an atmosphere with less than 0.1 ppm water and oxygen for stability test. It was found that the device with a conventional structure is intrinsically more stable than the device with inverted geometry under that same test condition. By minimizing oxygen and water content in the atmosphere for the duration of the lifetime test, the conventional device showed a much longer extrapolated lifetime of 20 years than the inverted device which showed the extrapolated lifetime of only 7 years.

### 4.3 Processing Strategies to Improve the Stability

The fabrication process of OSCs plays a significant role not only in the device performance but also in device stability. Many recent studies found that the optimization of the fabrication process can effectively enhance the stability of the device. During processing, many valid strategies are reported.

**Figure 25:**
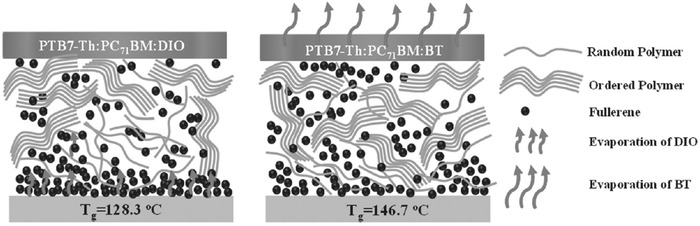
Morphology comparison between the DIO and BT added PTB7‐Th:PC71BM blends in the possessing. Reproduced with permission.^[^ ^265^ ^]^ Copyright 2017, John Wiley and Sons.

Optimizing the additives in the fabrication process of the active layer is one of the intensively studied methods in past 3 years. As mentioned, the widely used additive DIO is an unstable material which can decompose under the irradiation and cause instability in the device. Therefore, many studies focused on the design of alternative materials as additives to replace DIO in OSCs. Chen et al. reported the application of halogen‐free additive 1,4‐butanedithiol (BT) with low boiling point in the PTB7‐Th:PC_71_BM based device and compared it with the DIO.^[^ ^265^ ^]^ It was found that the replacement of DIO with BT can both enhance the performance and thermal stability of the device. They pointed out that additives with a low boiling point can create more thermally stable active layer. Similarly, Luscombe et al. revealed that the replacement of high boiling point additives with lower boiling additives could enhance the polymer stability in the active layer.^[^ ^266^ ^]^ They found that the additive with high boiling point is difficult to be entirely removed from the device during the fabrication process which can increase the oxygen diffusion to accelerate the photo‐oxidation. **Figure** **25** demonstrated the difference in the morphology and additives removing process between the DIO and BT. Later, Hou et al. designed a series of volatilizable solid additives (SA‐n) which can be removed easier compared to the DIO.^[^ ^267^ ^]^ As a result, the SA‐1 based device showed both enhanced PCE and light stability than the DIO based device. On the other hand, some additives can create a more stable morphology to enhance device stability. Yang et al. reported a novel polymer additive poly‐(pentafluorostyrene) (PPFS) and compared it with DIO in the PBDB‐TT5:ITIC based OSCs.^[^ ^268^ ^]^ They found that the PPFS can act as a morphological locking‐in agent in the active layer contributing to a more stable morphology. As a result, the PPFS based device showed both enhanced thermal stability and storage stability compared to DIO based device. Similarly, Hao et al. designed a novel small molecule based additive ethoxylated (2) bisphenol‐A dimethacrylate (BPA2EODMA) which can also act as the morphology locker in the active layer.^[^ ^145^ ^]^ Besides replacing the DIO, Moon et al. developed a co‐additives method to enhance device stability.^[^ ^269^ ^]^ They incorporated 4‐fluorobenzaldehyde (4‐FBD) with DIO as co‐additives in both PTB7:PC_71_BM and PBDB‐T:ITIC based OSCs and proved that the co‐additives based device could exhibit better air stability than the DIO based device when stored in ambient. The main reason was found that applying co‐additives can create a more stable interpenetrating morphology than the DIO.

Changing the solvent in the fabrication process of the active layer is another effective strategy to enhance device performance. Active layers cast from different solvent can show profound differences in the degradation process. Early in 2011, Yang et al. found that the P3HT:PC_61_BM based active layer cast from chlorobenzene (CB) can exhibit better light stability than the active layer cast 1,8‐octanedithiol (OT).^[^ ^270^ ^]^ Recently, many studies proved that the using of non‐halogenated solvent is a promising method toward stable OSCs. Roman et al. replaced the halogenated solvent o‐dichlorobenzene (o‐DCB) with the non‐halogenated solvent o‐MA in the PTB7‐Th:ITIC based OSCs.^[^ ^271^ ^]^ As results, the o‐methylanisole (o‐MA) processed device not only showed comparable PCE but also showed enhanced air stability when compared to the o‐DCB processed device under the ISOS‐D‐1 test condition. Jin et al. compared the solvent between halogenated CB and non‐halogenated toluene in OSCs.^[^ ^272^ ^]^ Similarly, the non‐halogenated toluene processed device showed enhanced air stability than the CB processed device. They revealed that nonhalogenated solvents could amend the quality of the morphology in the active layer which contributes to the enhanced long‐term stability in OSCs.

**Figure 26:**
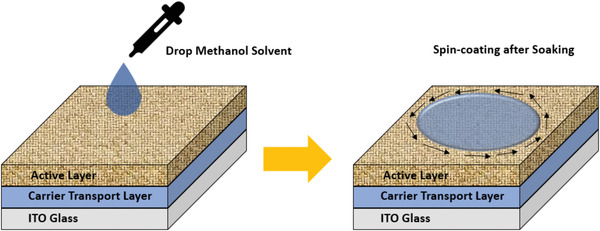
The schematic diagram of the active layer treated with the methanol solution in the fabrication process of OSCs. Reproduced with permission.^[^ ^275^ ^]^ Copyright 2017, Elsevier.

After the fabrication of the active layer, post‐treatments are usually applied to further enhance the performance of the device.^[^ ^27^, ^273^ ^]^ It is worth to mention that some post‐treatment strategies are also valid in enhancing device stability. For example, Uddin et al. proved that the thermal annealing treatment on the active layer could reduce the burn‐in degradation in the PBDB‐T:ITIC based devices.^[^ ^117^ ^]^ Samuel et al. proved that the thermal annealing and solvent annealing could contribute to better light stability in the SAM‐72:PC_71_BM based OSCs.^[^ ^274^ ^]^ They found that the more stable morphology in the active layer induced by annealing treatment can be the primary origin for the stability enhancement, as the annealed SAM‐72:PC_71_BM showed a fiber‐like structure with significantly enhanced film crystallinity. Besides the well‐established annealing strategy, solvent treatment is another newly developed strategy toward stable OSCs. Zhang et al. used methanol solvent to treat the as‐fabricated active layer for 2 min in the PTB7‐Th:PCDTBT:PC_70_BM based OSCs as shown in **Figure** **26**.^[^ ^275^ ^]^ It was proved that the methanol solvent treatment could effectively remove the residual additive DIO in the active layer and significantly enhance the performance and air stability of the device.

### 4.4 Encapsulation to Improve the Stability

Encapsulation is a well‐established strategy not only for OSCs but also for other types of solar cells to improve device stability. Uddin et al. have comprehensively reviewed the encapsulation strategy for both OSCs and perovskite solar cells.^[^ ^276^ ^]^ Encapsulating the device with organic, inorganic, and organic–inorganic complex materials can effectively prevent the device from exterior degradation factors such as oxygen and moisture.^[^ ^276^ ^]^ The encapsulation materials should have high a dielectric breakdown that matches the refractive index with other layers and high volume resistivity. Materials should be low cost, dimensionally stable, and easy to deposit. Some recent studies also reported that the encapsulation layer could act as the UV light blocker for OSCs to reduce UV degradation.^[^ ^72^ ^]^ Encapsulation layers with high quality can dramatically improve the device lifetimes. Moreover, the encapsulation can have an effect on the mechanical stability of OSCs. Using encapsulation materials with high flexibility can cause OSCs to have relatively higher mechanical stability.^[^ ^277^ ^]^ Highly flexible encapsulation materials can prevent the delamination and punctures in the encapsulation layer which create pathways for air penetration under the mechanical stress.^[^ ^276^, ^278^, ^279^ ^]^

## 5 Summary and Outlook

In this Review, we have discussed the limiting factors of stability of OSCs during their operation lifetime of metastable morphology, oxygen and water effects, heating and mechanical stress effects, diffusion of electrodes materials into the active layer. We have also highlighted the recent strategies to improve the stability of OSCs such as photo or photo/air stability, air stability, thermal and mechanical stability. The outlines of each stability strategies are as follows.

### Photo‐ or Photo/Air Stability

Improved the polymer crystallinity and reduced the fullerene crystallinity by employing cleavable side chains and photo/air‐stable units on the polymer molecules; reduced oxygen and moisture permeation to the active layer and reduced defects/traps in the active layer.

### Air Stability

Reduced oxygen and moisture permeation into the active layer by employing suitable electrode materials which have low reactivity with oxygen and moisture; employing hole and electron transport layers materials with inoxidizability, low hygroscopicity, and neutral PH value. Air stability is also improved by changing the device architecture.

### Thermal Stability

Reduced the crystallinity of fullerene materials and increase the glass transition temperature (*T*_g_) by employing a cross‐linkable polymer/fullerene or adding a cross‐linker. Thermal stability of OSCs is also improved by improving the active layer/electrode interfaces, forming a stable donor/acceptor (D/A) interface, and reduced residual solvents in the active layer.

### Mechanical Stability

Reduced polymer crystallinity and increased polymer flexibility and entanglement; improved layer/layer adhesion; forming a stable D/A interface and enhanced the flexibility of encapsulated materials.

For the commercial application in future, it is crucial to achieve higher efficiency and higher stability of OSCs. Some research area of device stability may need to focus in the future: i) design and synthesis of new polymer donor materials with balanced crystallinity/amorphism and rigidity/flexibility to simultaneously increase photo/air, thermal, and mechanical stabilities; ii) use non‐fullerene acceptors instead of PCBM for better photo/air, thermal, and mechanical stabilities; iii) in‐depth understanding of OSCs device degradation mechanisms with oxygen, water, irradiation, heating, and mechanical stress; iv) improve the PCE of OSCs on the basis of polymer with cleavable side chains and photo/air‐stable units; v) develop a uniform standard of OSCs stability tests, for example, ISOS protocols; vi) develop an advanced encapsulation technology in OSCs. The PCEs of OSCs has already exhibited over 16% and increasing the stability of OSCs will provide a great potential for future industrial manufacturing.

## Conflict of Interest

The authors declare no conflict of interest.
